# Supplementary material for: Genome wide identification of QTL associated with yield and yield components in two popular wheat cultivars TAM 111 and TAM 112
Source: PLoS One. 2020 Dec 2;15(12):e0237293. doi: 10.1371/journal.pone.0237293 (PMC7710072; doi:10.1371/journal.pone.0237293)
Supplement: S1 Fig — A. Boxplot analysis of yield and yield component traits. B. Dendrogram and biplot analysis of yield and yield component traits to classify mega-environments for each trait. (PDF) [file pone.0237293.s001.pdf]

S1a Fig: Boxplot Analysis of yield and yield component traits. Environments: Texas A&M AgriLife Research stations in Bushland, TX in 2011, 2012, 2017 as dryland (11BD, 12BD, 17BD), irrigated in 2017 (17BI), Chillicothe, TX in 2012 and 2014 as dryland (12CH and 14CH), and two irrigated levels (75% and 100%) in Etter, TX in 2013 and 2014 (13EP4, 13EP5, 14EP4 and 14EP5), Clovis, NM irrigated in 2017 (17CVI). Traits A) Plot yield from combine (YLD); B) dry biomass from hand harvested 0.5 m long inner row sample from crown (BM); C) grain weight from b) as hand harvested dry grain (BMYLD); D) harvest index (HI); E) kernels spike^{-1} (KPS); F) spikes m^{-2} (SPM); G) thousand kernel weight (TKW).

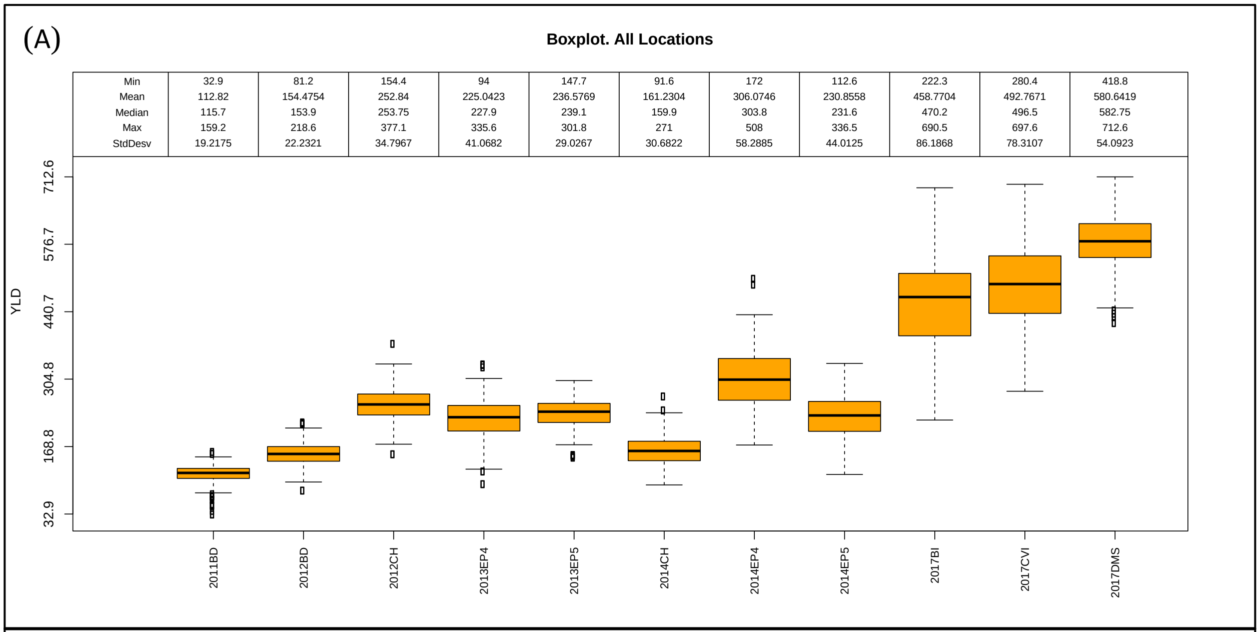

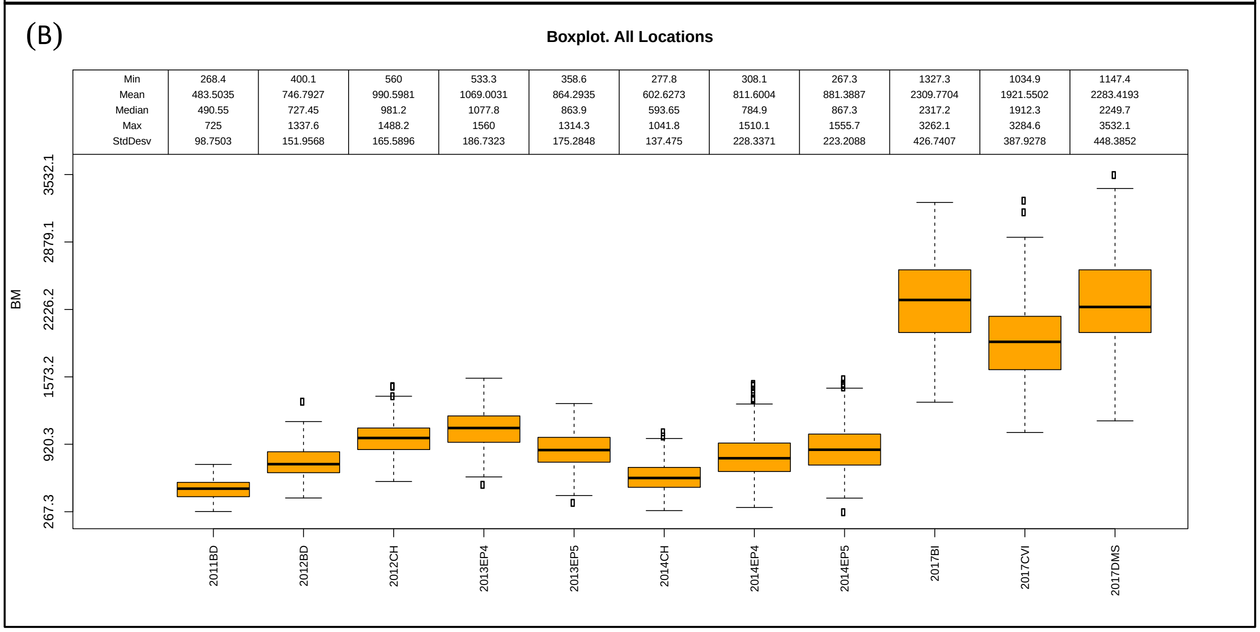

(C)

Boxplot. All Locations

Min	73.8	118.6	159	127.3	140.7	73	93.3	124.9	312.5	331.6	401
Mean	196.7637	260.4665	301.6804	301.89	284.5162	170.8766	334.728	359.0129	731.4289	663.8611	886.5277
Median	196.3	255.25	294.55	297.9	277.9	170.65	319.9	350.65	743.95	653.05	878.2
Max	312.7	483.4	502.1	507.8	454	310	687.5	651.5	1269.7	1126.7	1437.8
StdDesv	48.4978	64.4913	62.5726	70.2684	56.4289	45.8458	109.9229	100.2012	203.1394	165.846	188.2876

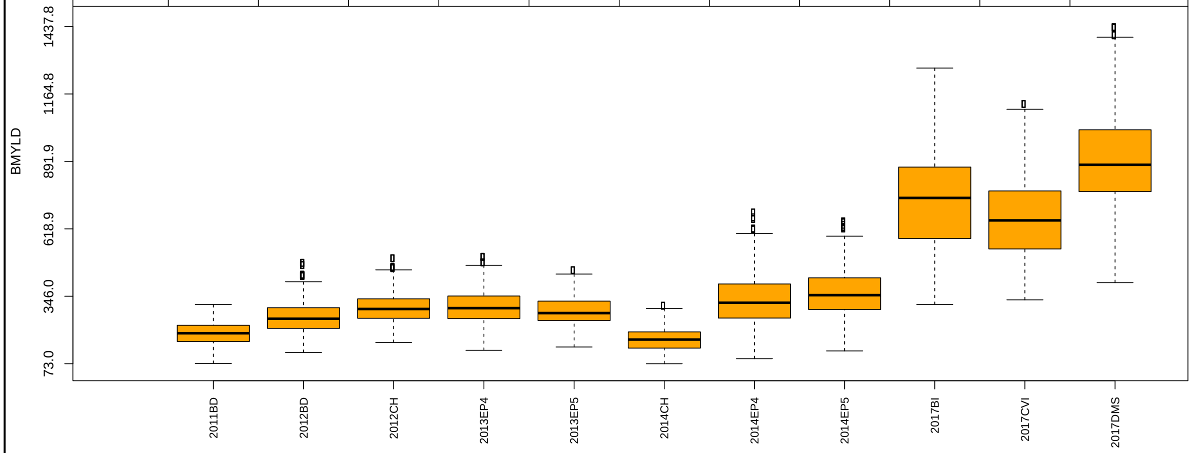

(D)

Boxplot. All Locations

Min	24	21	20	20	20	20	23	25	20.0188	21.1067	26.6045
Mean	40.7315	34.8654	30.572	28.1284	33.3203	28.477	41.3095	40.436	32.1095	34.6404	38.6256
Median	41	35	31	28	33	29	43	41	32.8737	34.8519	39.0714
Max	50	47	40	41	49	37	50	50	49.1286	49.2717	48.125
StdDesv	4.5063	4.6707	3.8512	3.9135	5.5043	4.0665	5.9076	5.7321	5.7121	4.3565	3.3797

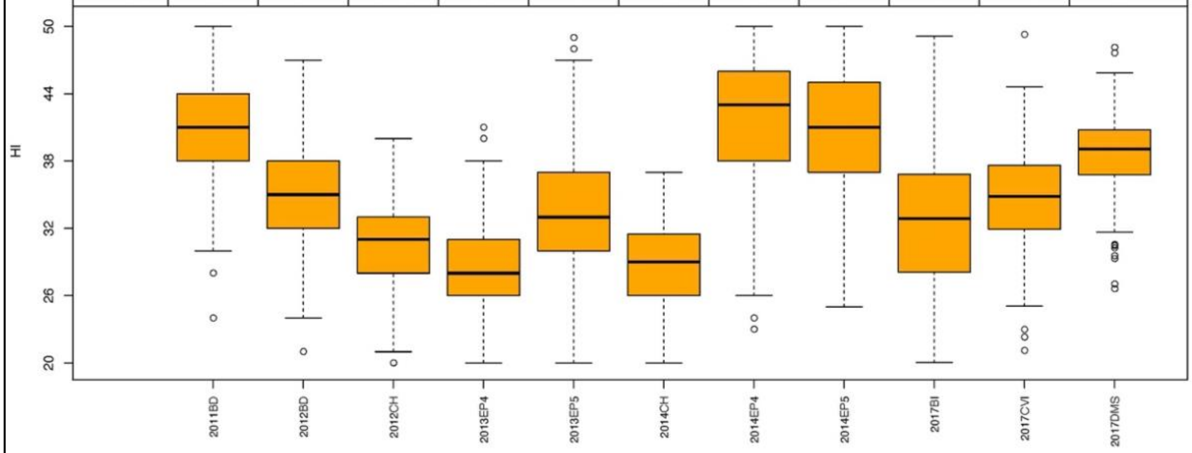

(E)

Boxplot. All Locations

Min	6.67	10.6	10.95	9.73	10.65	4.48	5.47	6.02	16.0221	10.1192	23.1644
Mean	17.8715	19.1544	20.5604	17.8352	18.6978	15.1041	18.685	18.3361	26.2256	19.8932	32.3514
Median	18.03	18.96	20.39	17.6	18.265	15.42	18.84	18.22	26.4325	19.8843	32.5709
Max	29.86	43.5	36.58	27.16	45.38	23.03	30.43	34.22	41.5218	29.1542	43.0511
StdDesv	3.8751	3.6441	3.3593	3.1	3.8976	3.5587	4.4451	4.2715	5.5206	3.1794	3.8055

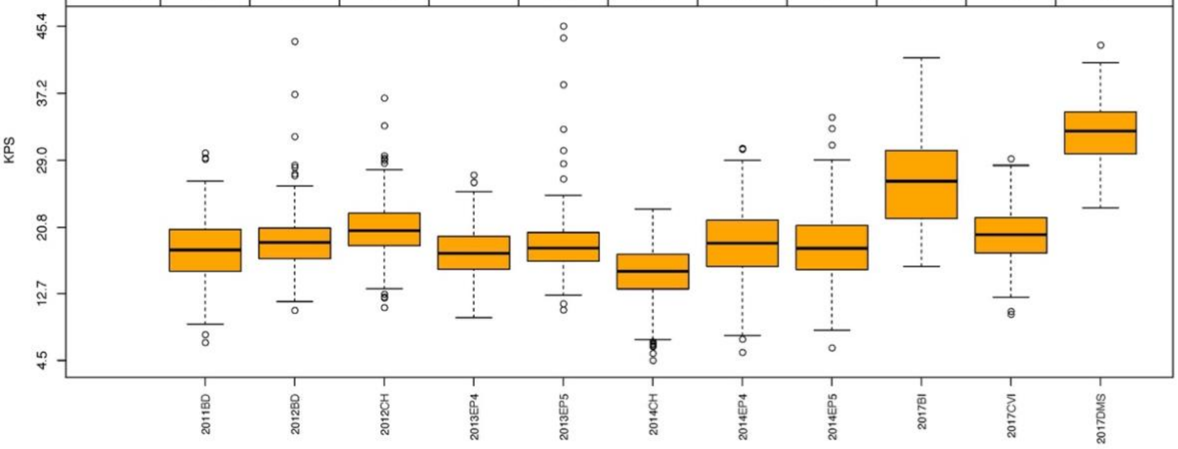

(F)

Boxplot. All Locations

Min	224.97	348.71	371.2	359.96	258.72	292.46	314.96	292.46	416.198	404.9494	449.9438
Mean	462.6809	638.401	667.3295	864.1436	713.5068	503.4617	571.2808	623.8212	850.3152	995.7394	838.4998
Median	461.19	629.92	663.67	843.64	697.41	494.94	551.18	618.67	854.8931	978.6277	832.396
Max	821.15	1102.36	967.38	1484.81	1169.85	832.4	1034.87	1068.62	1259.8425	1631.0461	1226.0967
StdDesv	103.658	127.2174	112.6709	181.7048	153.4197	93.309	140.5595	152.9645	153.6064	209.5224	157.3646

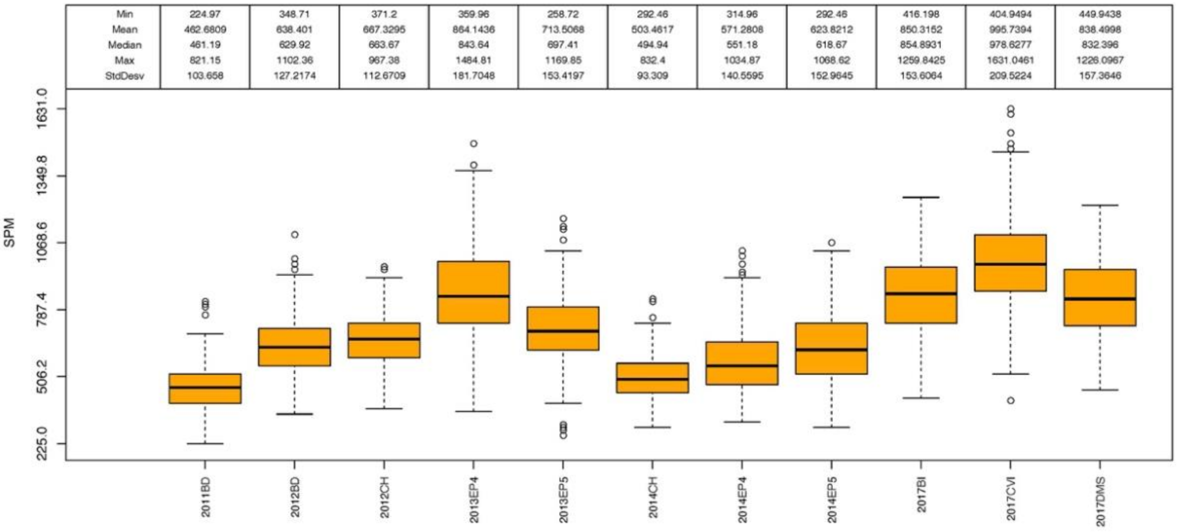

(G)

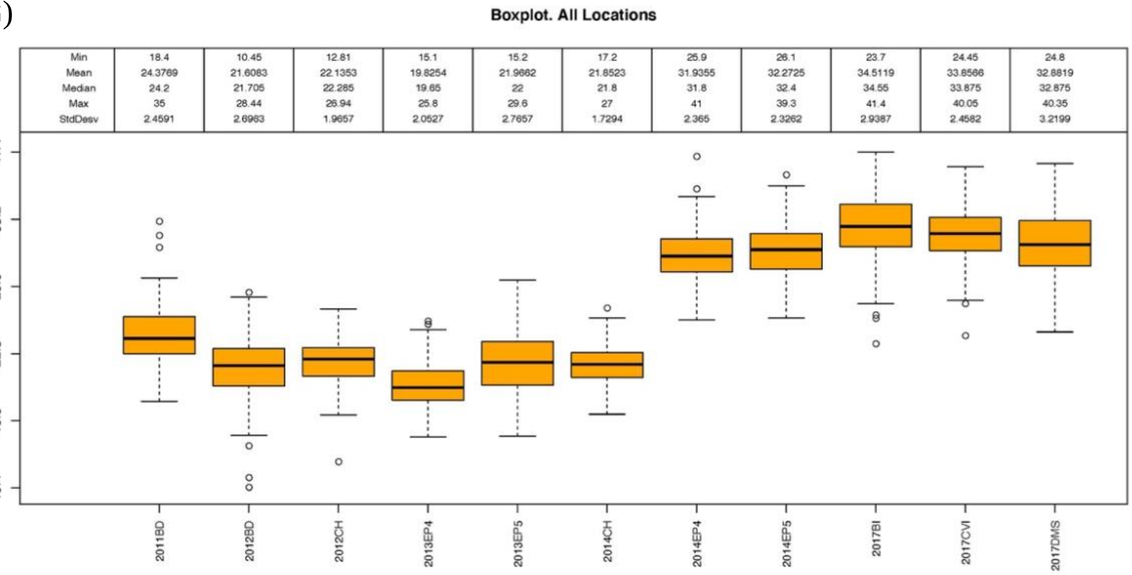
Boxplot. All Locations

	2011BD	2012BD	2012CH	2013EP4	2013EP5	2014CH	2014EP4	2014EP5	2017BI	2017CVI	2017DMS
Min	18.4	10.45	12.81	15.1	15.2	17.2	25.9	26.1	23.7	24.45	24.8
Mean	24.3769	21.6083	22.1353	19.8254	21.9662	21.8523	31.9355	32.2725	34.5119	33.8566	32.8819
Median	24.2	21.705	22.285	19.65	22	21.8	31.8	32.4	34.55	33.875	32.875
Max	35	28.44	26.94	25.8	29.6	27	41	39.3	41.4	40.05	40.35
StdDesv	2.4591	2.6963	1.9657	2.0527	2.7657	1.7294	2.365	2.3262	2.9387	2.4582	3.2199

TKW
41.4
35.2
29.0
22.8
16.6
10.4
2011BD
2012BD
2012CH
2013EP4
2013EP5
2014CH
2014EP4
2014EP5
2017BI
2017CVI
2017DMS

S1b Fig: Dendrogram and biplot Analysis of yield and yield component traits to classify mega-environments for each trait. Environments: Texas A&M AgriLife Research stations in Bushland, TX in 2011, 2012, 2017 as dryland (11BD, 12BD, 17BD), irrigated in 2017 (17BI), Chillicothe, TX in 2012 and 2014 as dryland (12CH and 14CH), and two irrigated levels (75% and 100%) in Etter, TX in 2013 and 2014 (13EP4, 13EP5, 14EP4 and 14EP5), Clovis, NM irrigated in 2017 (17CVI). Mega-environment (ME) MEs were as follow: YLD: ME1(17BI, 17CVI, 14EP4, 14EP5), ME2(12BD, 13EP4, 13EP5), ME3(12CH, 14CH); BM: ME1 (11BD, 14CH, 14EP4), ME2 (13EP4, 13EP5); BMYLD: ME1 (11BD, 14CH), ME2 (13EP4, 12BD, 12CH), ME3 (14EP4, 14EP5, 17CVI); HI: ME1(14EP5, 17BI, 17CVI, 17DMS), ME2(12CH, 14CH, 13EP4), ME3(12BD, 11BD, 13EP5); KPS: ME1(17CVI, 17DMS, 14EP4, 14EP5), ME2(11BD, 12BD, 13EP4, 14CH); SPM: ME1(14EP5, 14CH), ME2(12BD, 13EP4, 13EP5, 17CVI), ME3(12CH, 17DMS); TKW: ME1(11BD, 12BD, 12CH, 13EP4, 13EP5, 14CH), ME2(14EP4, 14EP5, 17BI, 17CVI, 17DMS). Traits A) Yield from combine plots (YLD); B) dry biomass from hand harvested 0.5 m long inner row sample from crown (BM); C) grain weight from B) as hand harvested dry grain (BMYLD); D) harvest index (HI); E) kernels spike^{-1} (KPS); F) spikes m^{-2} (SPM); G) thousand kernel weight (TKW).

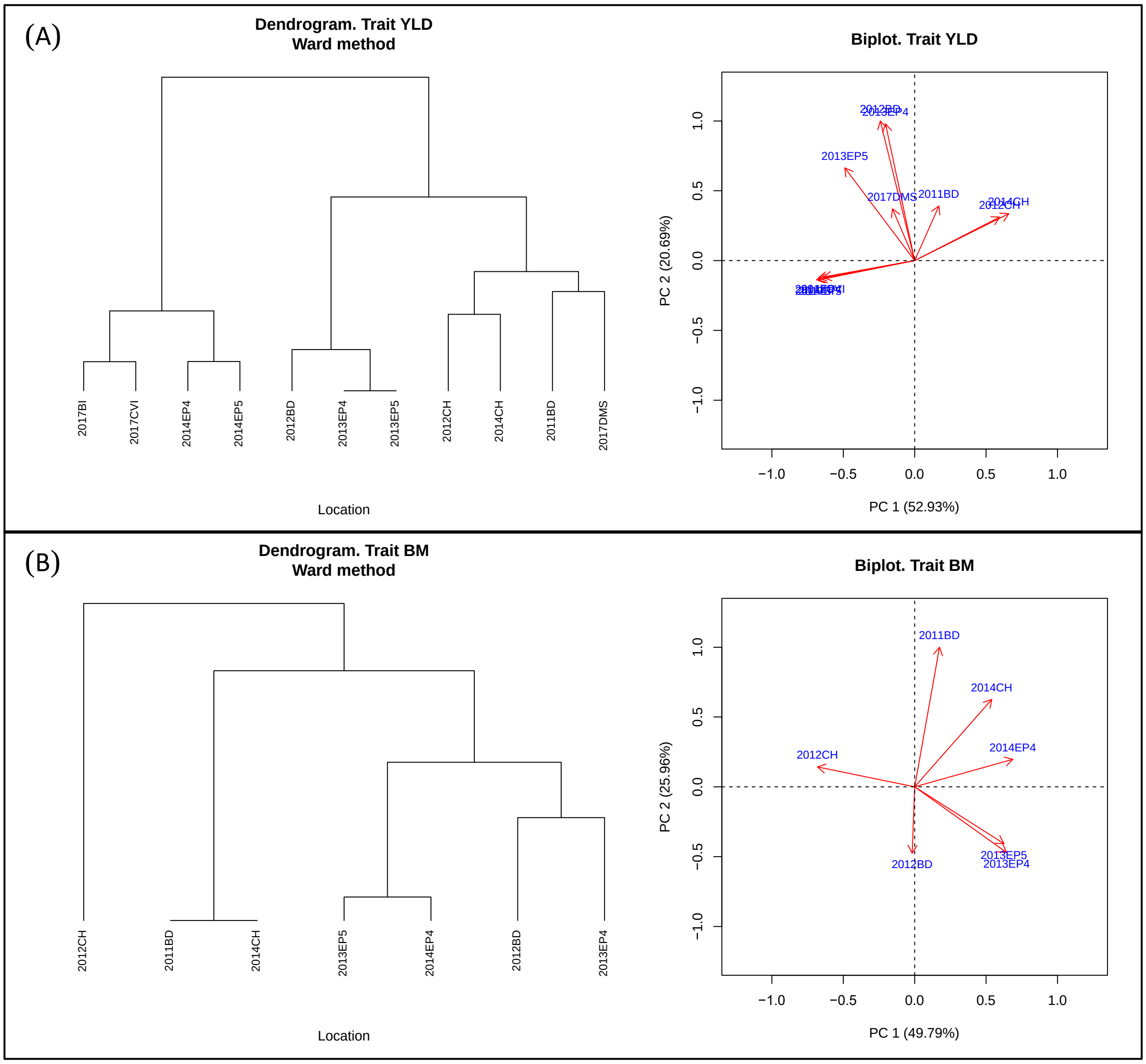

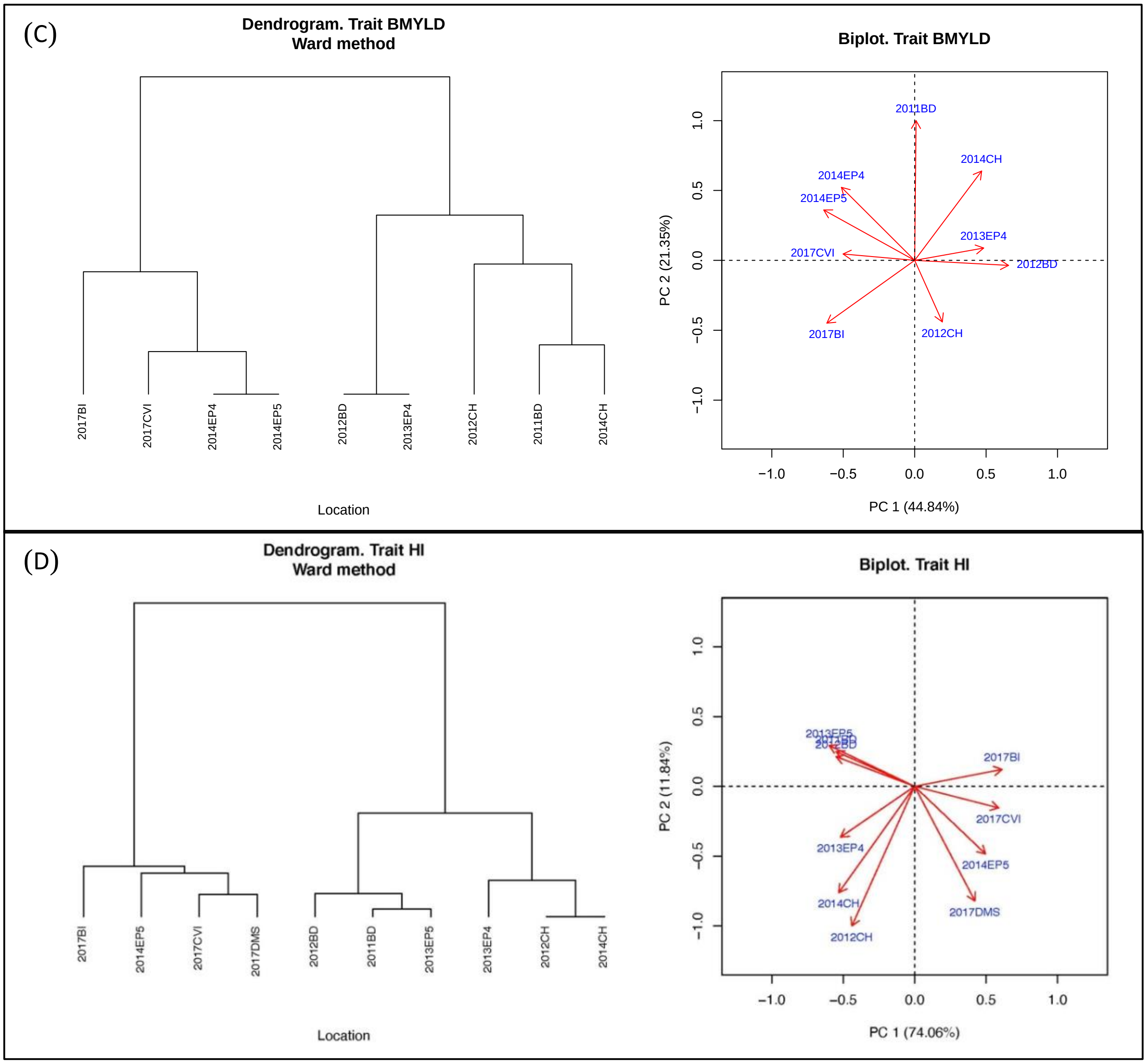
(C)
Dendrogram. Trait BMYLD
Ward method
2017BI
2017CVI
2014EP4
2014EP5
2012BD
2013EP4
2012CH
2011BD
2014CH
Location
Biplot. Trait BMYLD
2011BD
2014CH
2014EP4
2014EP5
2013EP4
2017CVI
2012BD
2017BI
2012CH
PC 2 (21.35%)
PC 1 (44.84%)
−1.0
−0.5
0.0
0.5
1.0
(D)
Dendrogram. Trait HI
Ward method
2017BI
2014EP5
2017CVI
2017DMS
2012BD
2011BD
2013EP5
2013EP4
2012CH
2014CH
Location
Biplot. Trait HI
2013EP5
2012BD
2017BI
2017CVI
2013EP4
2014EP5
2014CH
2017DMS
2012CH
PC 2 (11.84%)
PC 1 (74.06%)
−1.0
−0.5
0.0
0.5
1.0

(E)

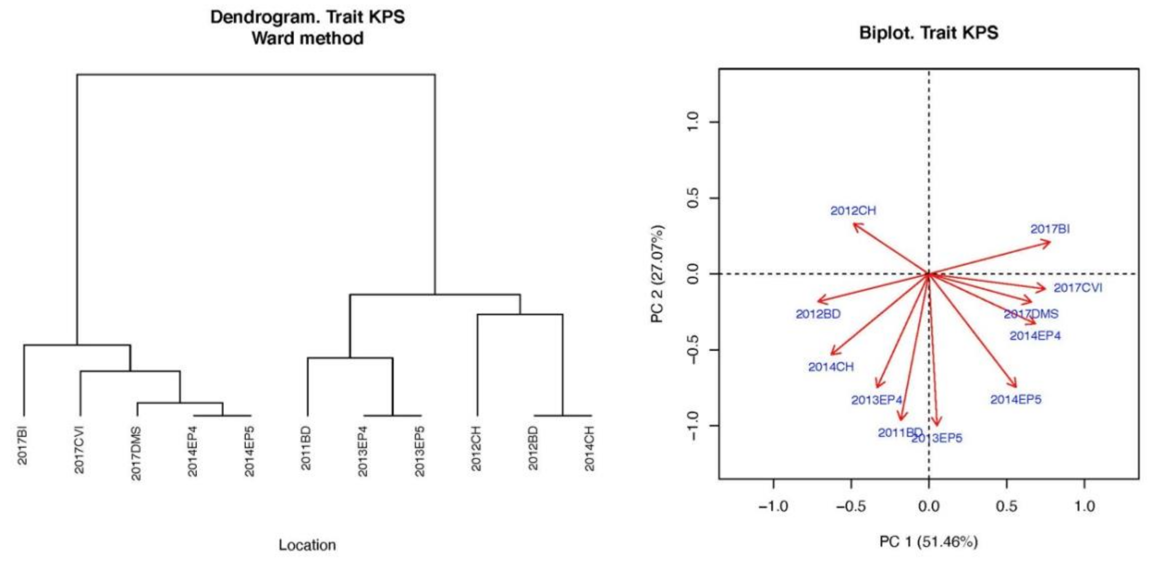
Dendrogram. Trait KPS
Ward method
2017BI
2017CVI
2017DMS
2014EP4
2014EP5
2011BD
2013EP4
2013EP5
2012CH
2012BD
2014CH
Location
Biplot. Trait KPS
PC 2 (27.07%)
PC 1 (51.46%)
2012CH
2017BI
2017CVI
2012BD
2017DMS
2014EP4
2014CH
2013EP4
2014EP5
2011BD
2013EP5

(F)

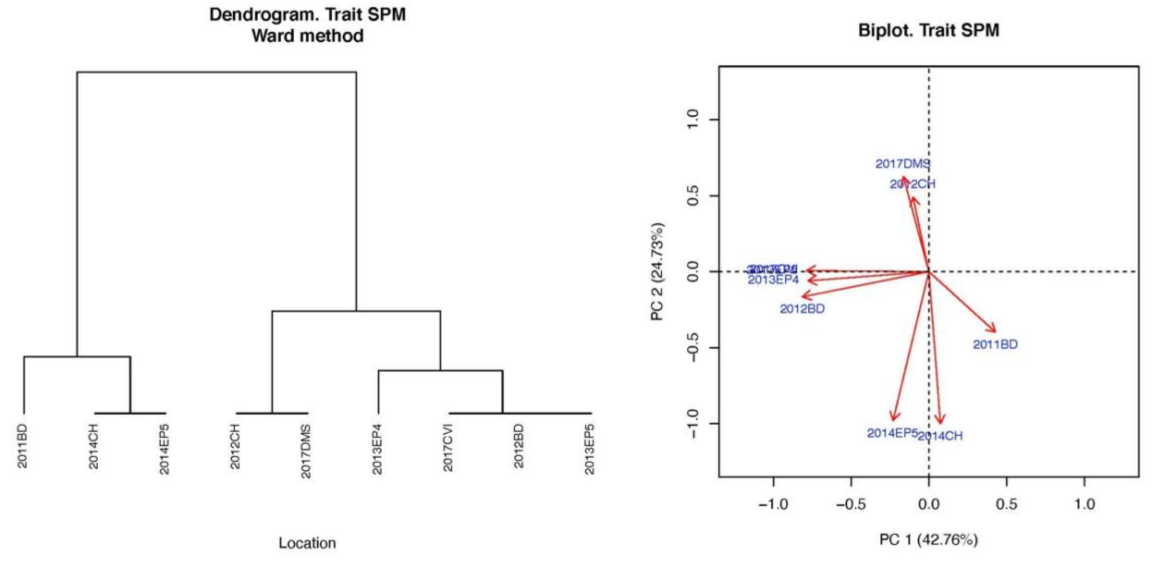
Dendrogram. Trait SPM
Ward method
2011BD
2014CH
2014EP5
2012CH
2017DMS
2013EP4
2017CVI
2012BD
2013EP5
Location
Biplot. Trait SPM
PC 2 (24.73%)
PC 1 (42.76%)
2017DMS
2012CH
2013EP4
2012BD
2011BD
2014EP5
2014CH

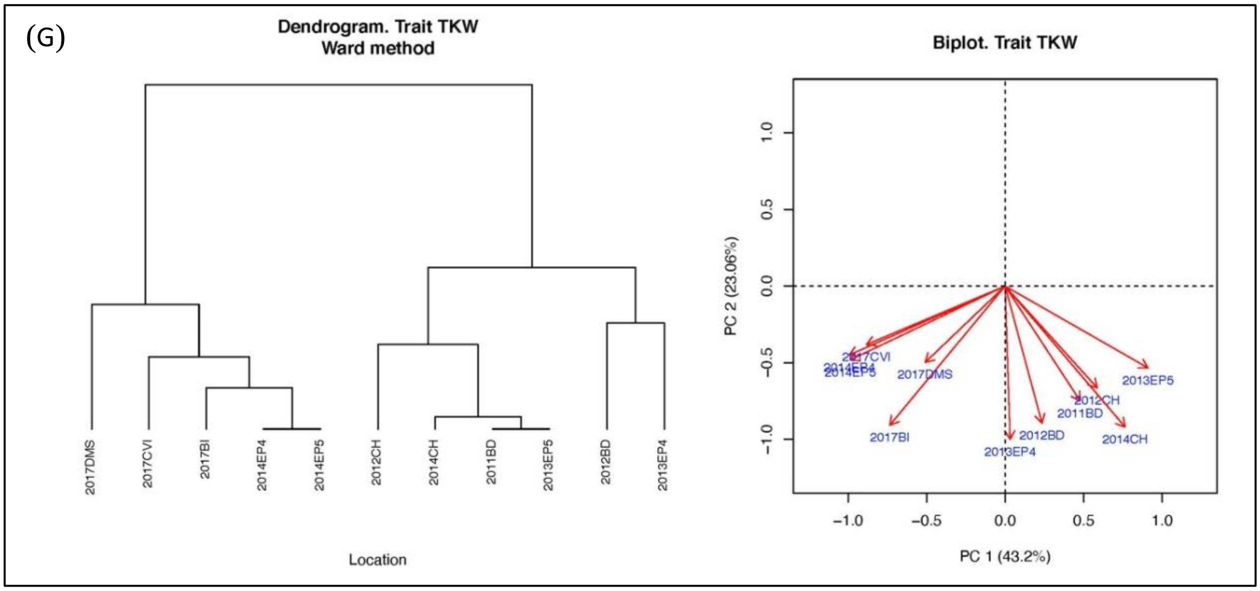
(G)
Dendrogram. Trait TKW
Ward method
2017DMS
2017CVI
2017BI
2014EP4
2014EP5
2012CH
2014CH
2011BD
2013EP5
2012BD
2013EP4
Location
Biplot. Trait TKW
PC 2 (23.06%)
PC 1 (43.2%)
2017CVI
2014EP4
2014EP5
2017DMS
2017BI
2013EP5
2012CH
2011BD
2012BD
2013EP4
2014CH
