# Supplementary material for: Genome wide identification of QTL associated with yield and yield components in two popular wheat cultivars TAM 111 and TAM 112
Source: PLoS One. 2020 Dec 2;15(12):e0237293. doi: 10.1371/journal.pone.0237293 (PMC7710072; doi:10.1371/journal.pone.0237293)
Supplement: S2 Fig — (PDF) [file pone.0237293.s002.pdf]

S2 Fig: LOD profile and additive effects of QTLs detected in each of the 11 environments. Top figure shows LOD profile with chromosomal position along the x-axis (cM of 25 LGs) and LOD score on the y-axis. Bottom figure show additive effect profile with chromosomal position along the x-axis and additive effects on the y-axis. Different yield and yield components traits are color coded as shown on the top of each panel. The positive additive effect means that the favorable alleles increasing the traits were from TAM 112 while those QTL with negative additive effects had favorable alleles from TAM 111. Traits include 1) Yield from combine plots (CombYld=Yld); 2) dry biomass from hand harvested 0.5 m long inner row sample from crown (DBM=BM); 3) grain weight from 2) as hand harvested dry grain weight (HHDG=BMYLD); 4) harvest index (HI); 5) kernels spike^{-1} (KPS); 6) spikes m^{-2} (SPM); 7) thousand kernel weight (TKW); 8) single head dry weight (SHDW); 9) single head dry grain weight (SHGW). Individual environments include A) 11BD, B) 12BD, C) 12CH, D) 13EP4, E) 13EP5, F) 14CH, G) 14EP4, H) 14EP5, I) 17BI, J) 17CVI, K) 17DMS

(A)

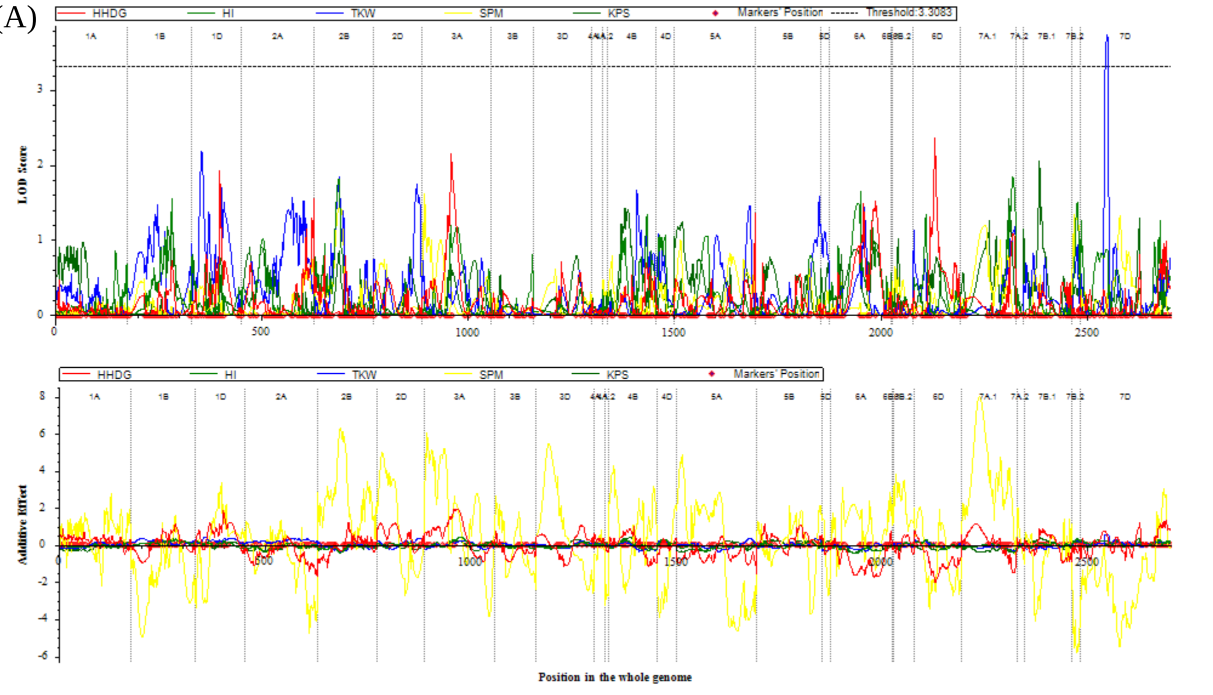

(B)

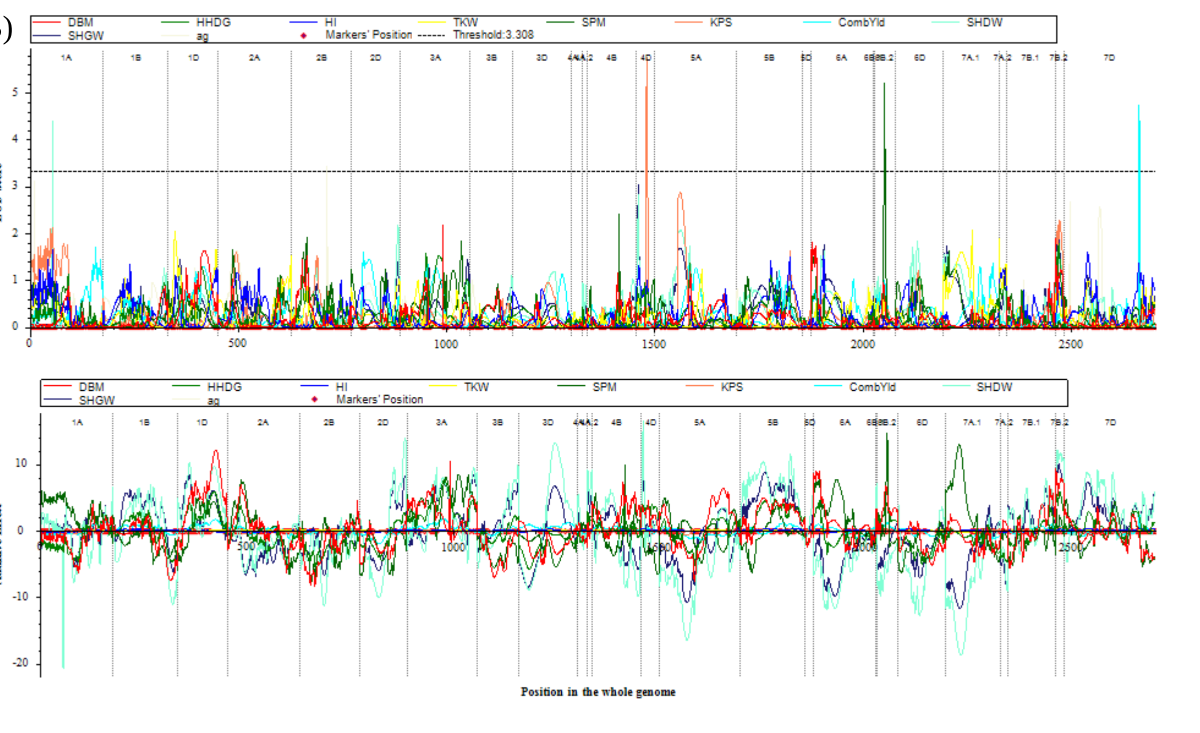
DBM
HHDG
HI
TKW
SPM
KPS
CombYld
SHDW
SHGW
ag
Markers' Position
Threshold:3.308
LOD Score
Additive Effect
Position in the whole genome

(C)

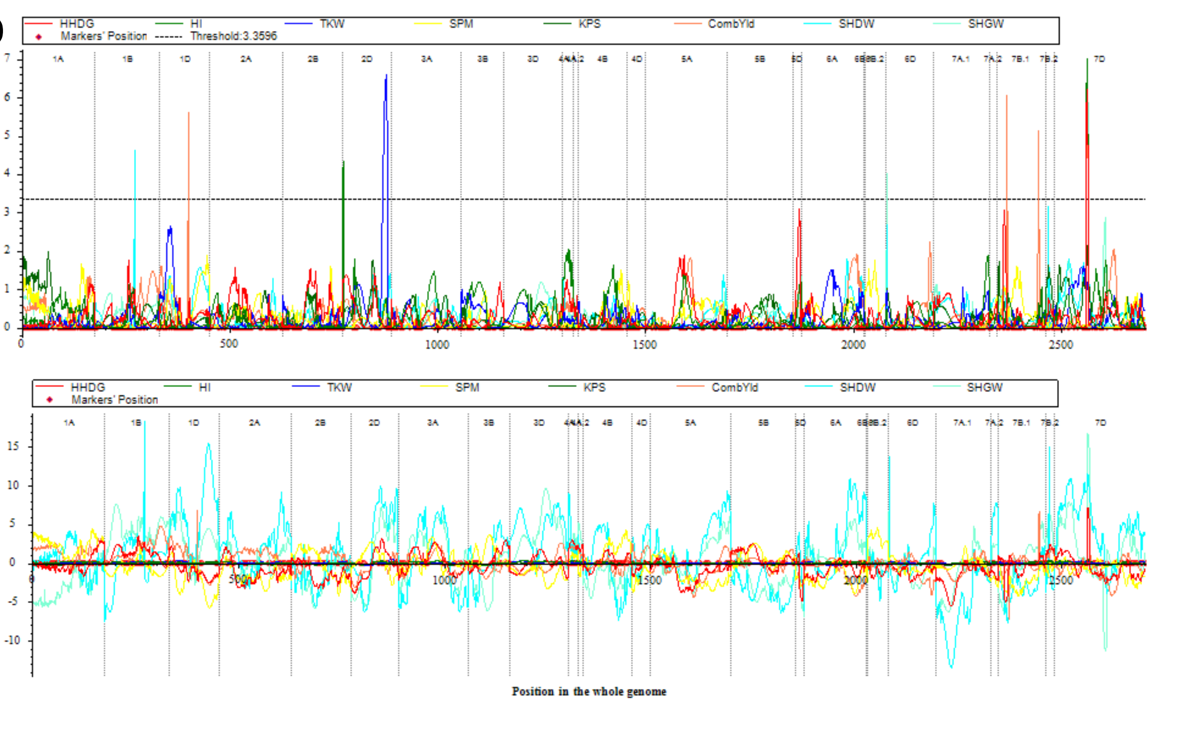
HHDG
HI
TKW
SPM
KPS
CombYld
SHDW
SHGW
Markers' Position
Threshold:3.3596
LOD Score
Additive Effect
Position in the whole genome

(D)

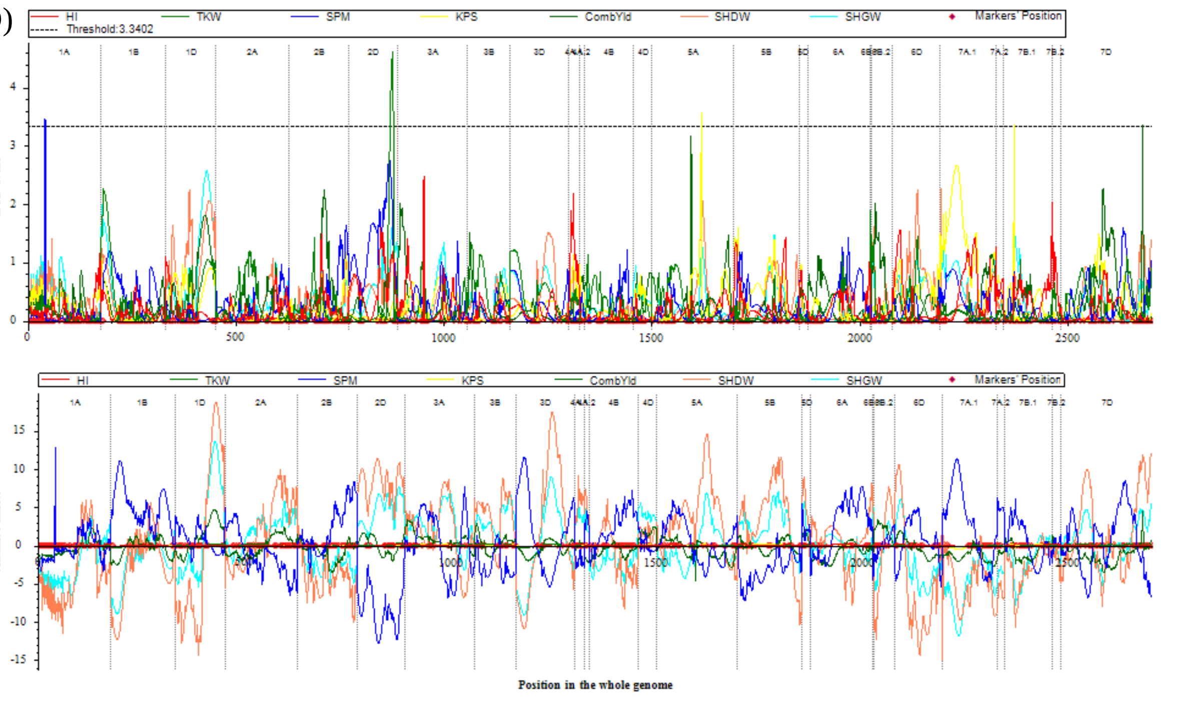
HI
TKW
SPM
KPS
CombYld
SHDW
SHGW
Markers' Position
Threshold:3.3402
1A 1B 1D 2A 2B 2D 3A 3B 3D 4A 4B 4D 5A 5B 5D 6A 6B 6D 7A.1 7A.2 7B.1 7B.2 7D
LOD Score
Additive Effect
Position in the whole genome

(E)

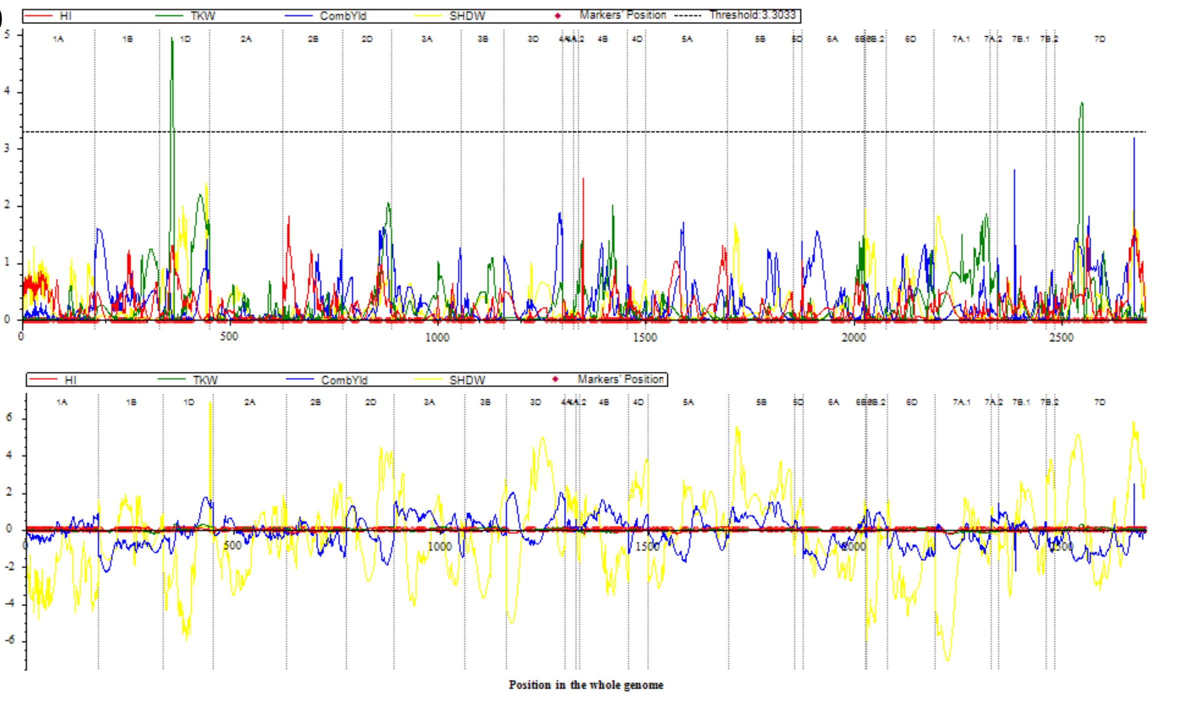
HI
TKW
CombYld
SHDW
Markers' Position
Threshold:3.3033
1A 1B 1D 2A 2B 2D 3A 3B 3D 4A 4B 4D 5A 5B 5D 6A 6B 6D 7A.1 7A.2 7B.1 7B.2 7D
LOD Score
Additive Effect
Position in the whole genome

(F)

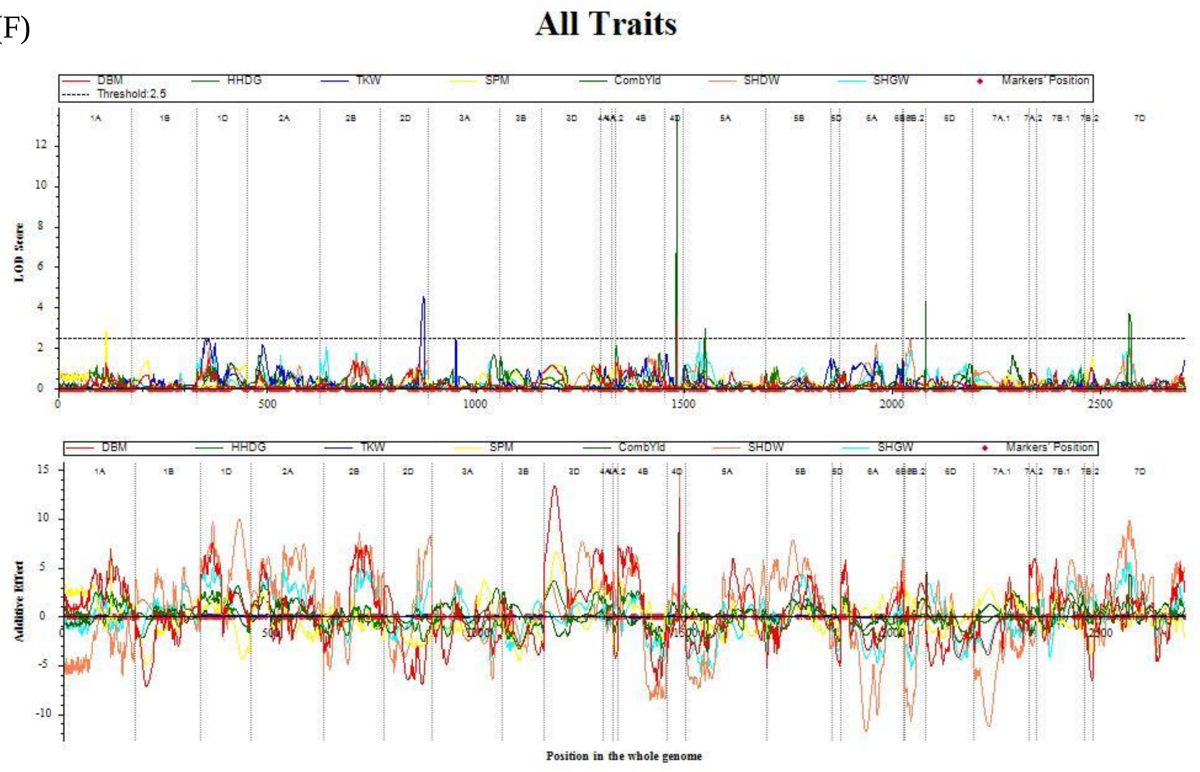
All Traits
DBM
HHDG
TKW
SPM
CombYld
SHDW
SHGW
Markers' Position
Threshold:2.5
LOD Score
Additive Effect
Position in the whole genome

(G)

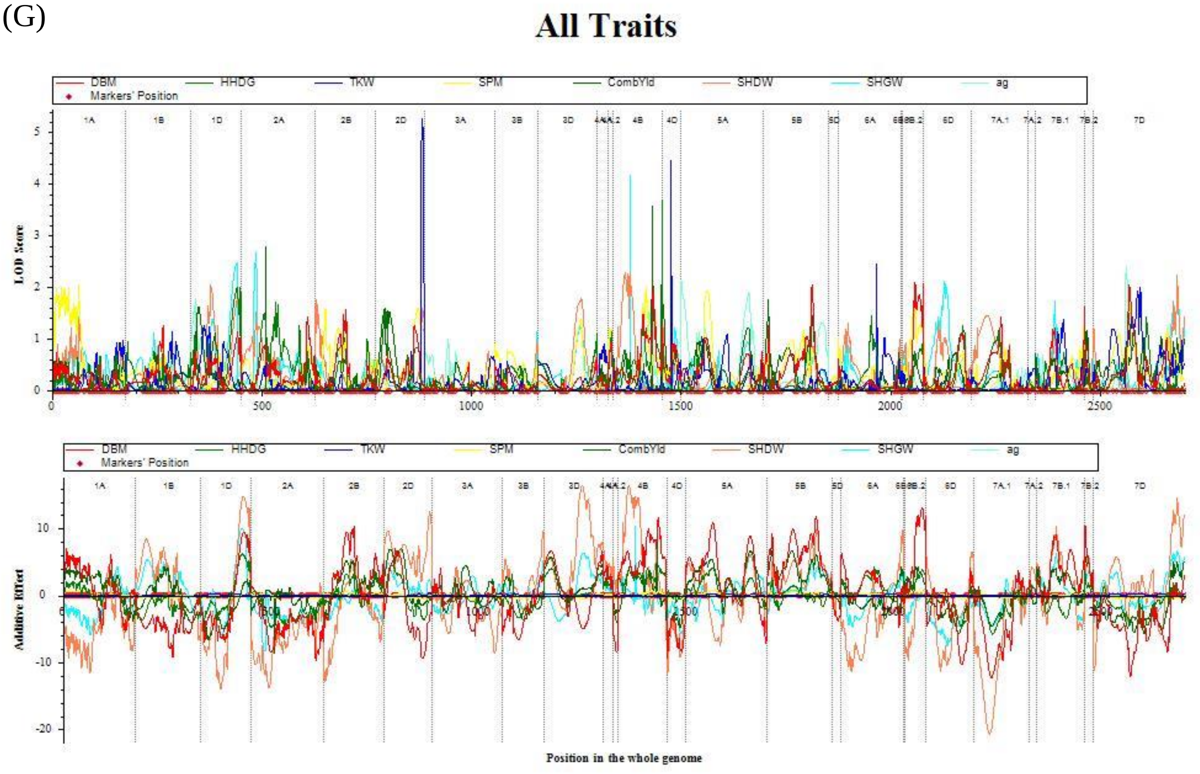
All Traits
DBM
HHDG
TKW
SPM
CombYld
SHDW
SHGW
ag
Markers' Position
LOD Score
Additive Effect
Position in the whole genome

(H)

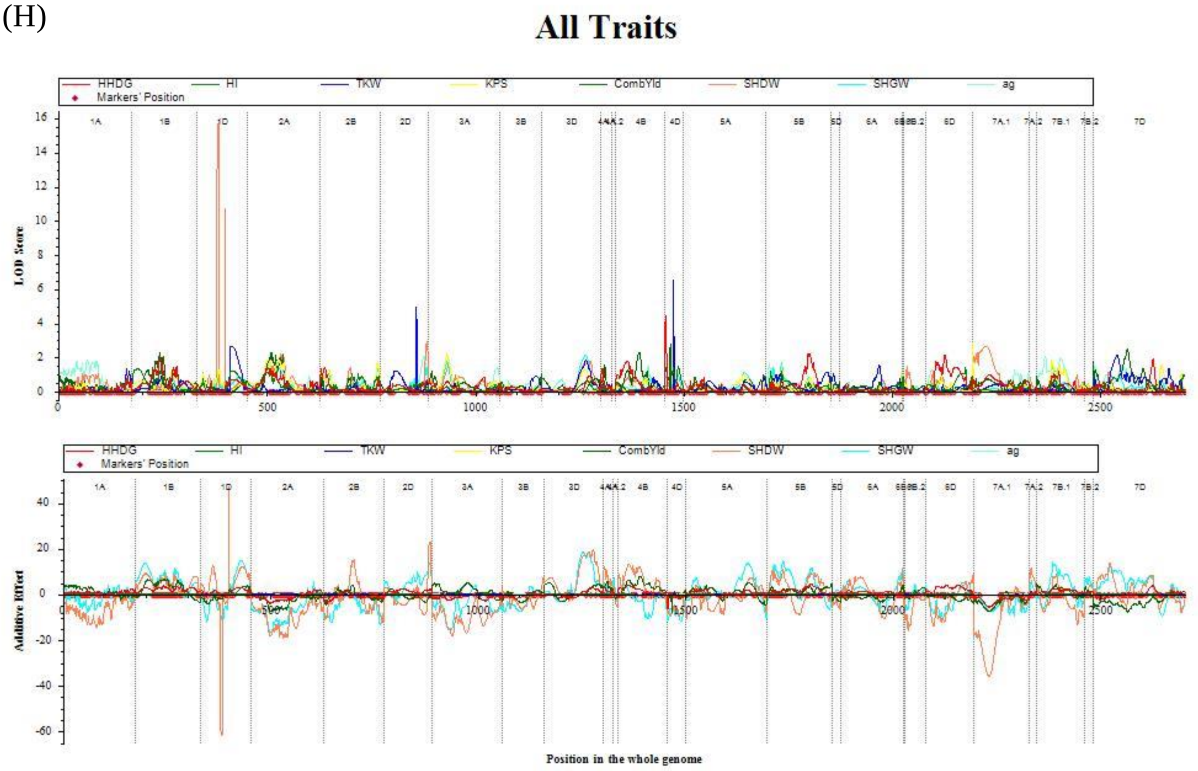
All Traits
HHDG
HI
TKW
KPS
CombYld
SHDW
SHGW
ag
Markers' Position
LOD Score
Additive Effect
Position in the whole genome

(I)

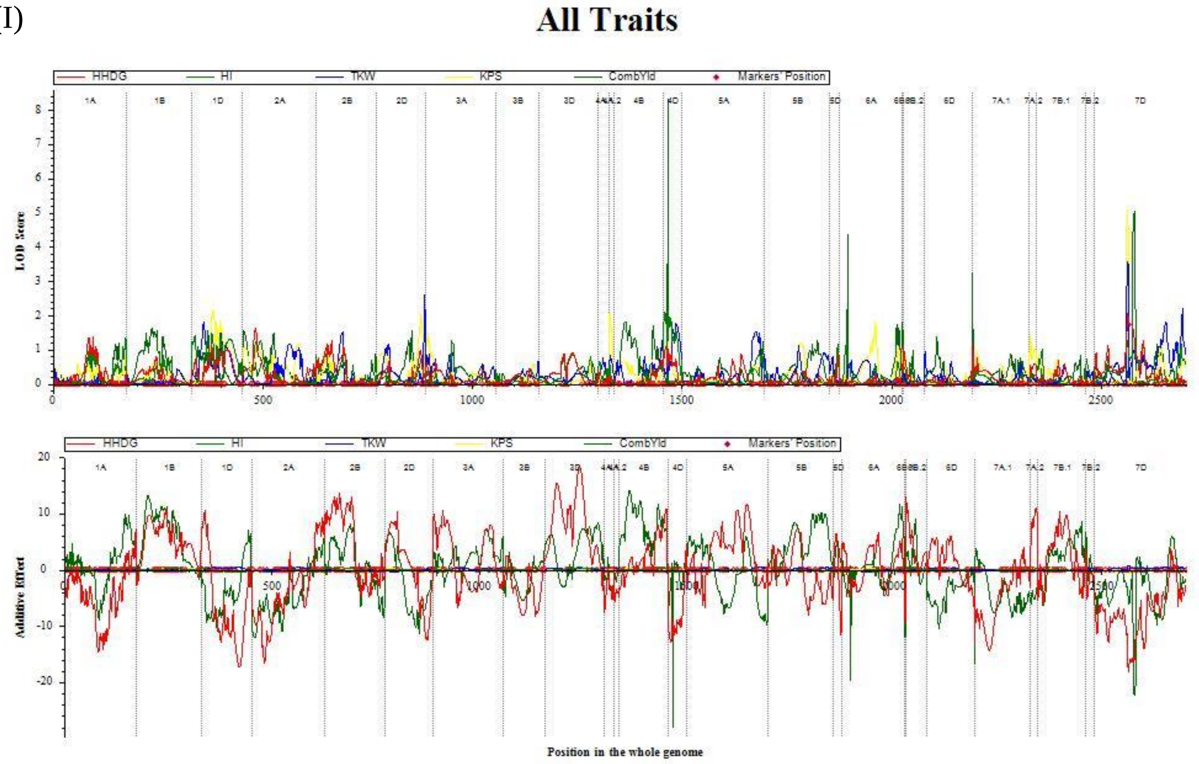
All Traits
HHDG
HI
TKW
KPS
CombYld
Markers' Position
LOD Score
Additive Effect
Position in the whole genome

(J)

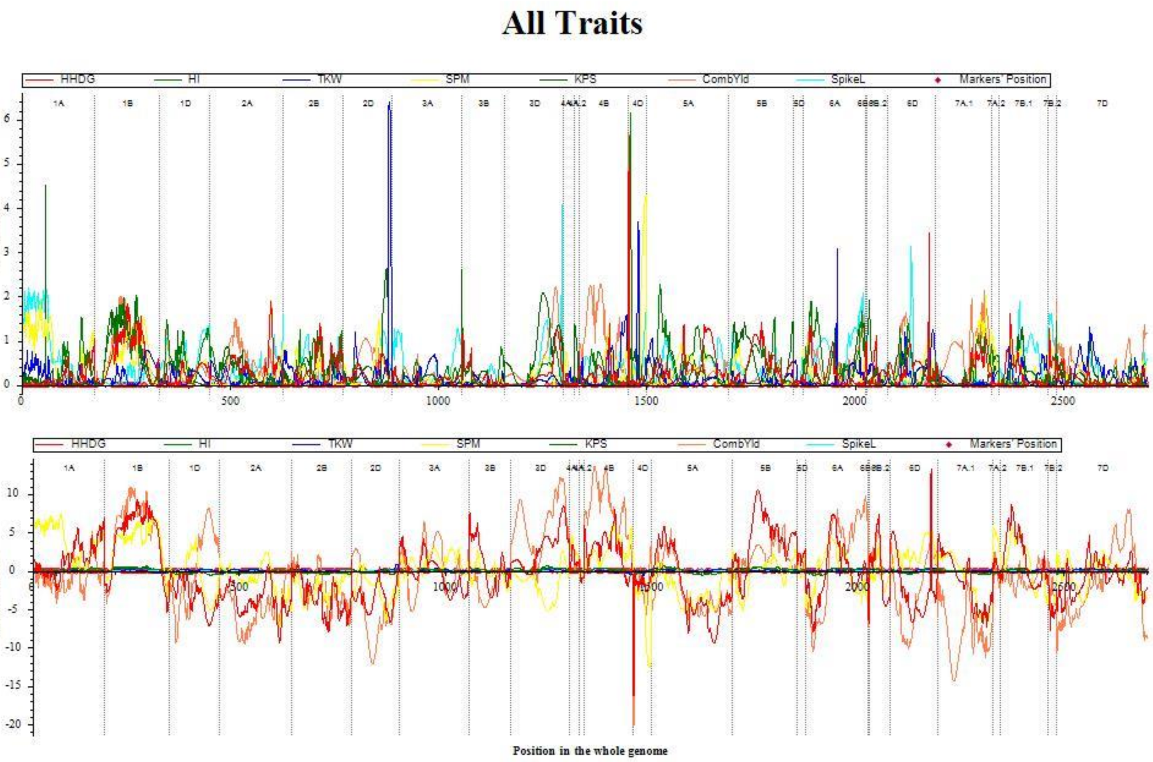
All Traits
HHDG
HI
TKW
SPM
KPS
CombYld
SpikeL
Markers' Position
LOD Score
Additive Effect
Position in the whole genome

(K)

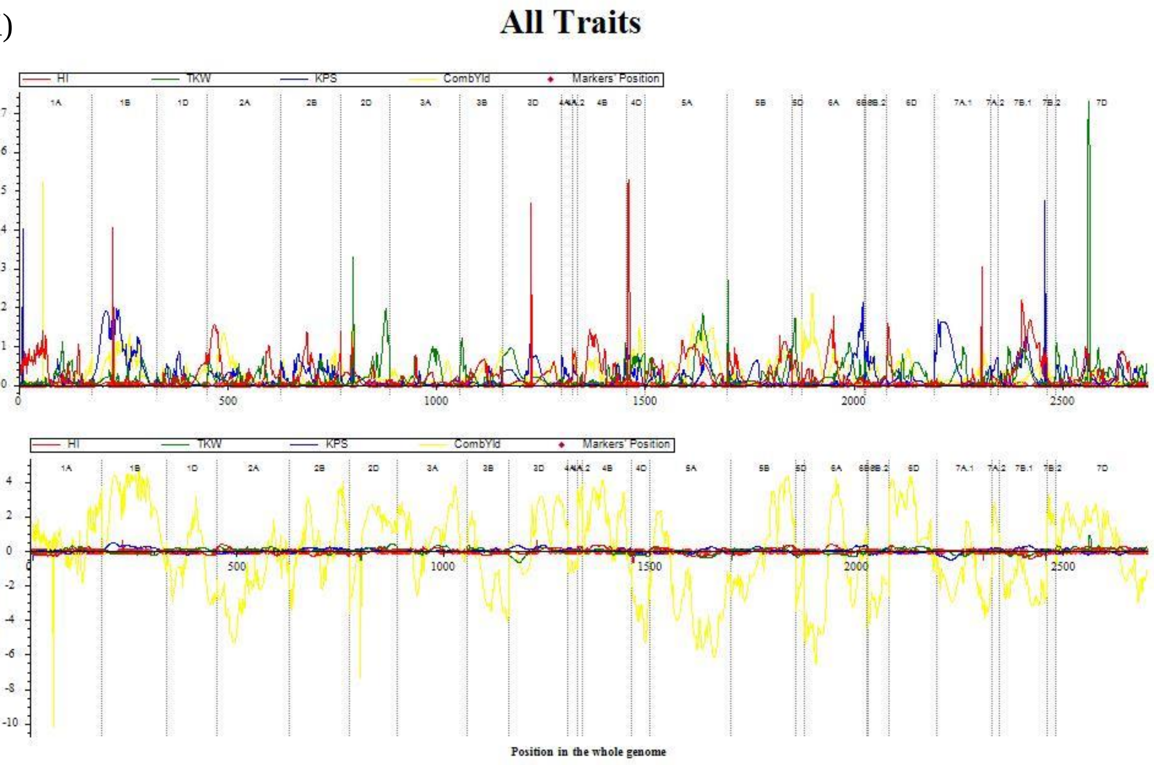
All Traits
HI
TKW
KPS
CombYld
Markers' Position
LOD Score
Additive Effect
Position in the whole genome
