# Supplementary material for: Genome wide identification of QTL associated with yield and yield components in two popular wheat cultivars TAM 111 and TAM 112
Source: PLoS One. 2020 Dec 2;15(12):e0237293. doi: 10.1371/journal.pone.0237293 (PMC7710072; doi:10.1371/journal.pone.0237293)
Supplement: S3 Fig — (PDF) [file pone.0237293.s003.pdf]

S3 Fig. Whole genome significance LOD(A) and LOD(AbyE) profiles of quantitative trait loci for yield and its components based on across all the environments for each trait. LOD for additive effect LOD(A) is in red, LOD scores of additive by environment LOD(AbyE) is in green; the dot line paralleled the y-axis separated the 25 linkage groups while the x-axis from left to right are the total length of cM from chromosome 1A to 7D matching the 25 linkage groups (Table S3). The additive effect above the zero line in the bottom figure means that the favorable alleles increasing the traits were from TAM 112 while those QTL below had favorable alleles from TAM 111. Traits include A) Yield from combine plots (YLD); B) dry biomass from hand harvested 0.5 m long inner row sample from crown (BM); C) grain weight from b) as hand harvested dry grain (BMYLD); D) harvest index (HI); E) kernels spike^{-1} (KPS); F) spikes m^{-2} (SPM); G) thousand kernel weight (TKW).

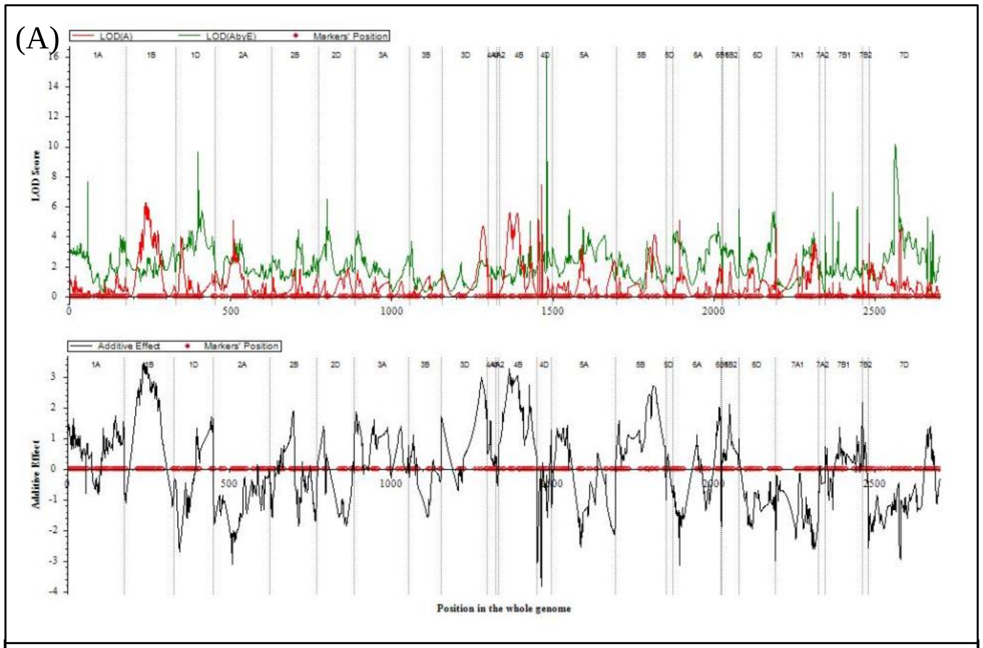

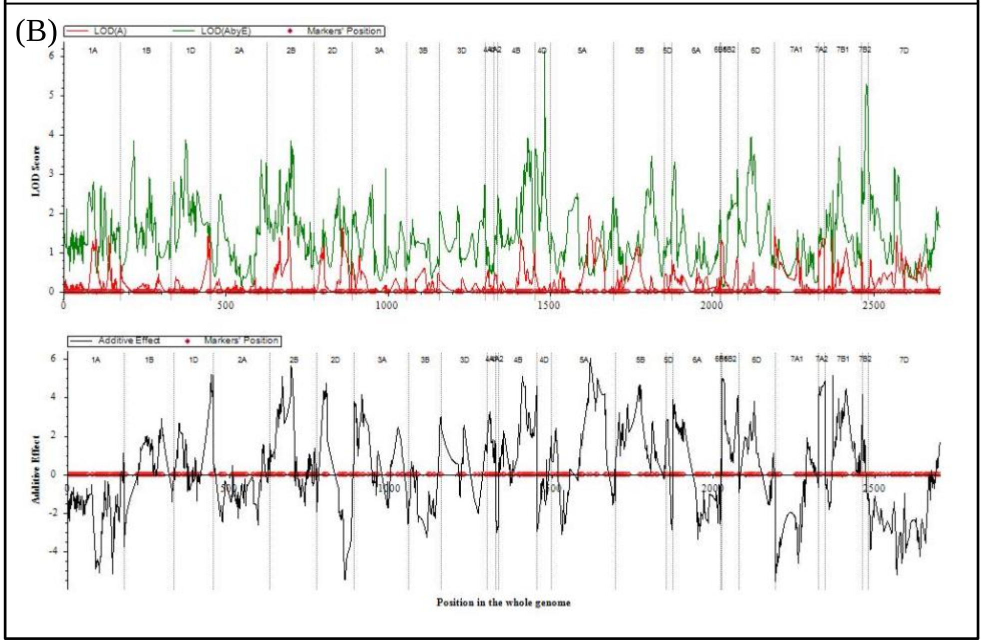

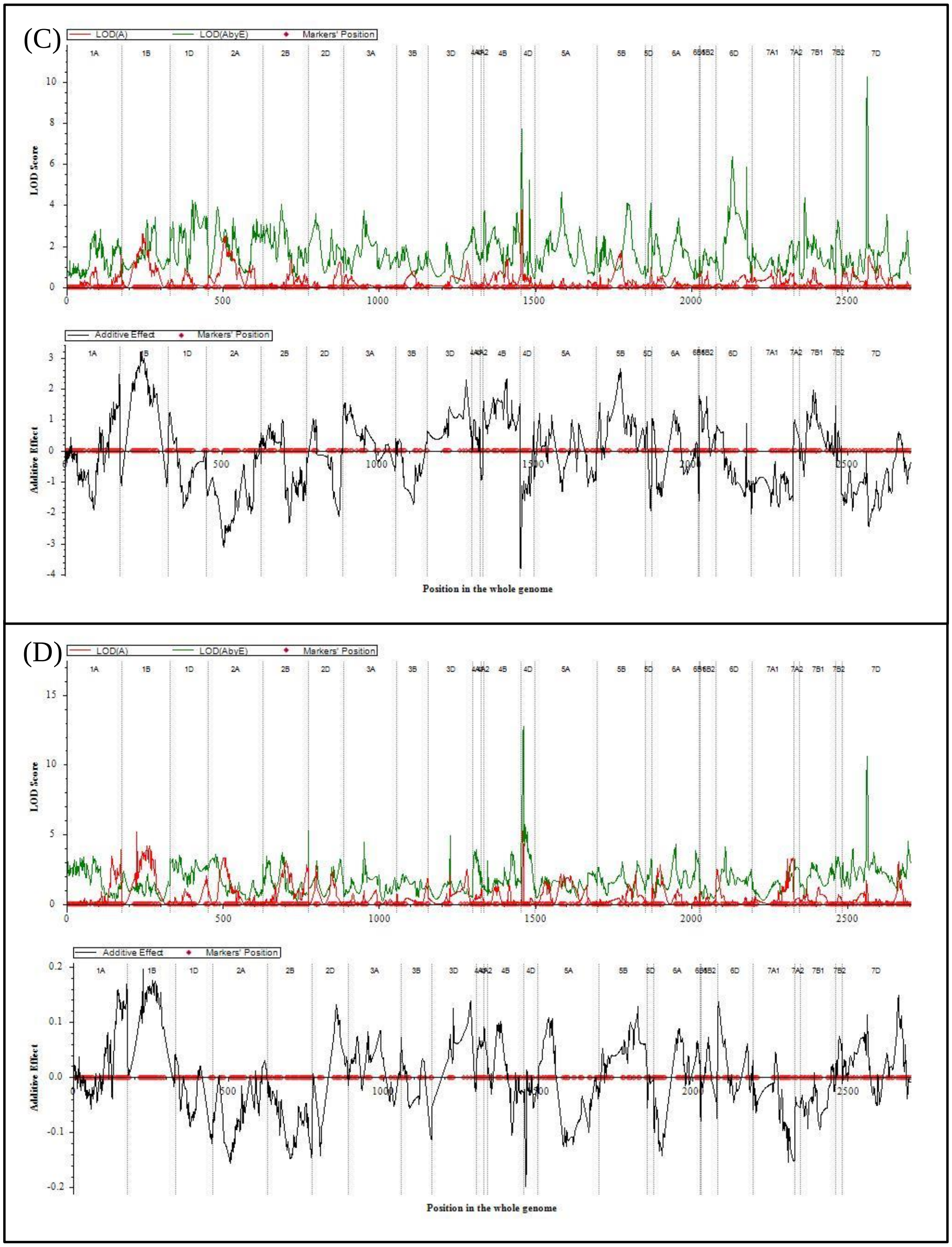
(C)
LOD(A)
LOD(AbyE)
Markers' Position
1A 1B 1D 2A 2B 2D 3A 3B 3D 4B 4D 5A 5B 5D 6A 6D 7A1 7A2 7B1 7B2 7D
LOD Score
Additive Effect
Position in the whole genome
(D)
LOD(A)
LOD(AbyE)
Markers' Position
LOD Score
Additive Effect
Position in the whole genome

(E)

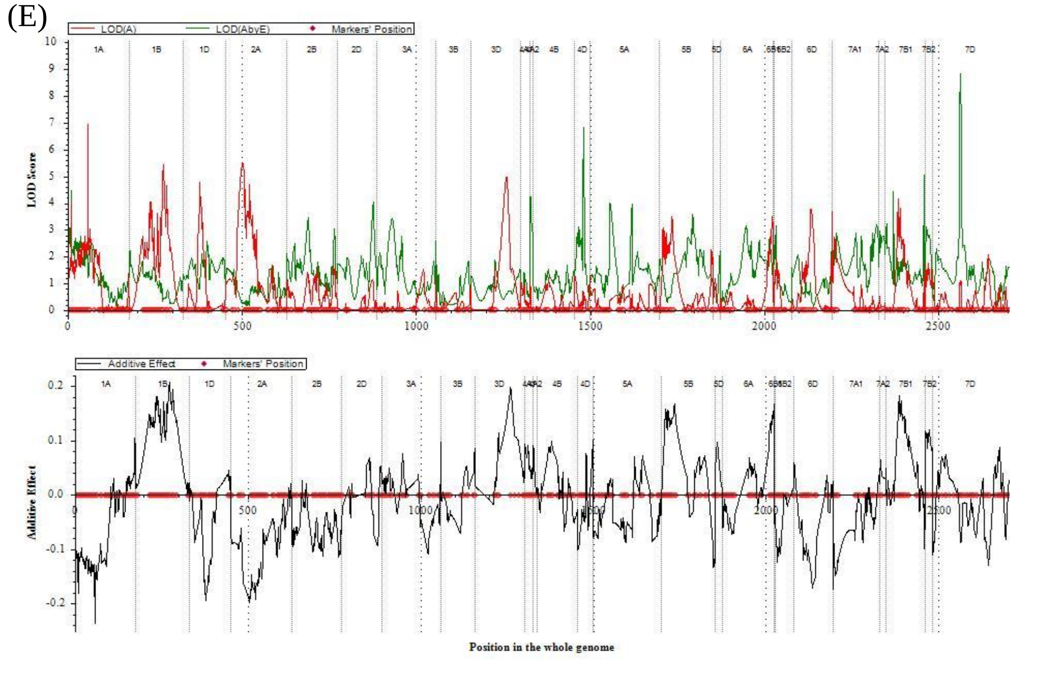
LOD(A)
LOD(AbyE)
Markers' Position
LOD Score
1A 1B 1D 2A 2B 2D 3A 3B 3D 4B 4D 5A 5B 5D 6A 6D 7A1 7A2 7B1 7B2 7D
Additive Effect
Position in the whole genome

(F)

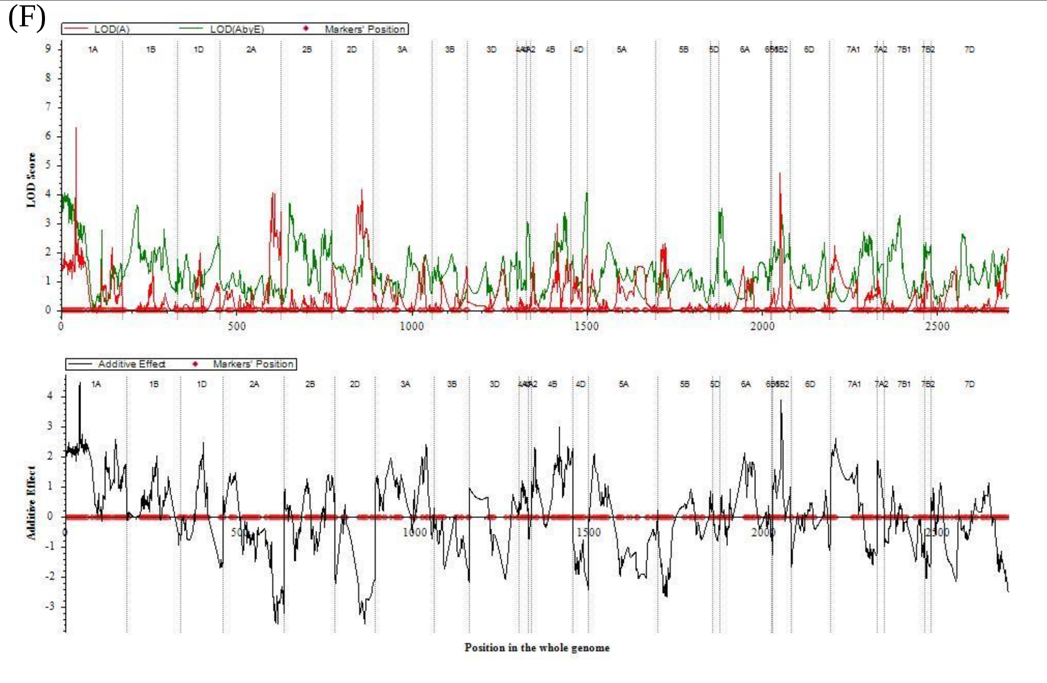
LOD(A)
LOD(AbyE)
Markers' Position
LOD Score
1A 1B 1D 2A 2B 2D 3A 3B 3D 4B 4D 5A 5B 5D 6A 6D 7A1 7A2 7B1 7B2 7D
Additive Effect
Position in the whole genome

(G)

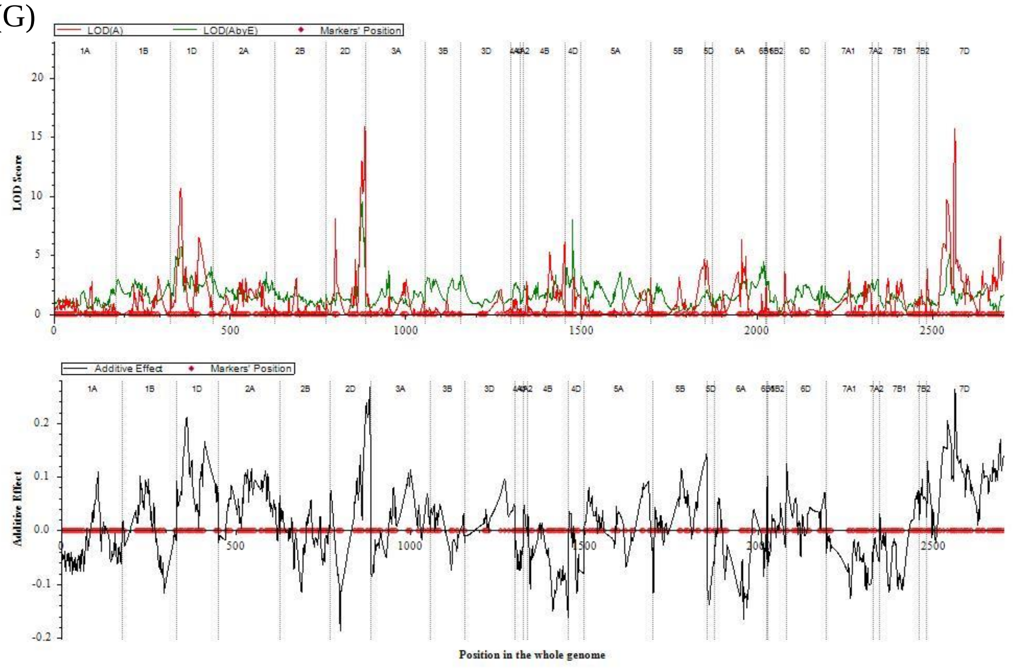

LOD(A)
LOD(AbyE)
Markers' Position
LOD Score
Additive Effect
Position in the whole genome
