# Supplementary material for: Genome wide identification of QTL associated with yield and yield components in two popular wheat cultivars TAM 111 and TAM 112
Source: PLoS One. 2020 Dec 2;15(12):e0237293. doi: 10.1371/journal.pone.0237293 (PMC7710072; doi:10.1371/journal.pone.0237293)
Supplement: S4 Fig — (PDF) [file pone.0237293.s004.pdf]

S4 Fig. QTL allele effects on spike m^{-2} and kernels spike^{-1} from various environemnts with the presence of significance of QTL. Total six QTL combinations were classified as 21 groups with number of RILs from two to ten. The means of each trait and their corresponding standard deviations were showed in the graphs.

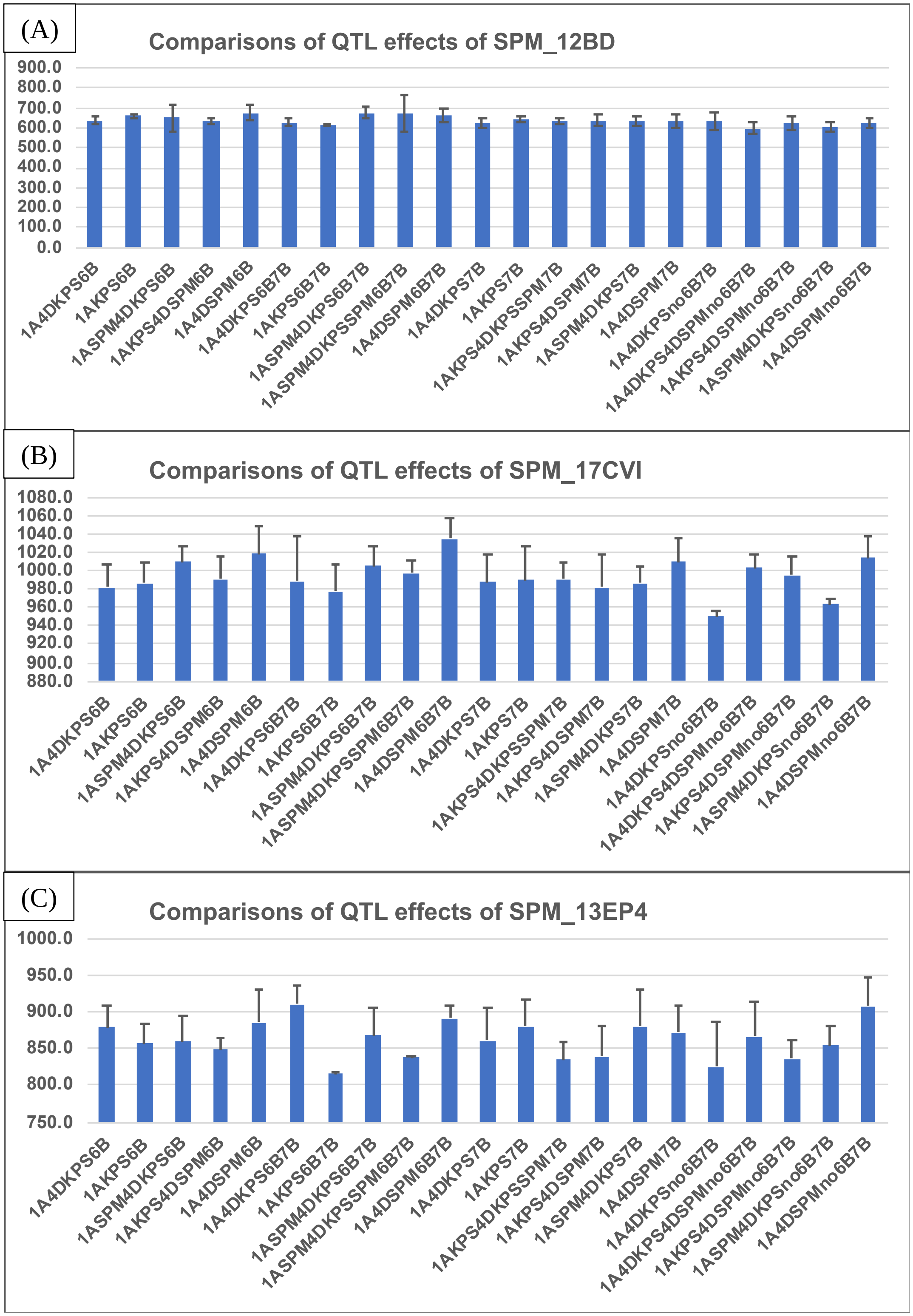

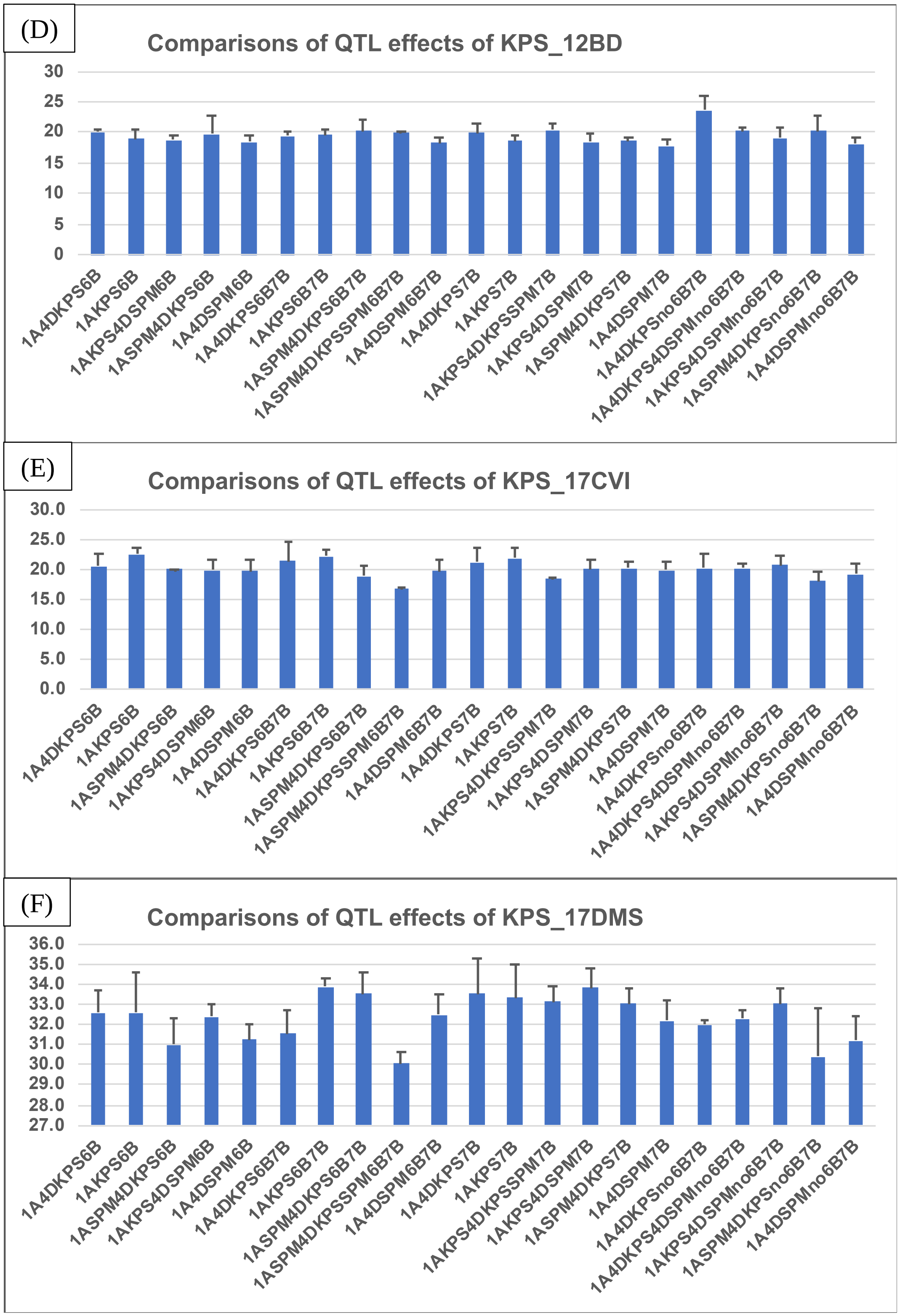
(D)
Comparisons of QTL effects of KPS_12BD
30
25
20
15
10
5
0
1A4DKPS6B
1AKPS6B
1AKPS4DSPM6B
1ASPM4DKPS6B
1A4DSPM6B
1A4DKPS6B7B
1AKPS6B7B
1ASPM4DKPS6B7B
1ASPM4DKPSSPM6B7B
1A4DSPM6B7B
1A4DKPS7B
1AKPS7B
1AKPS4DKPSSPM7B
1AKPS4DSPM7B
1ASPM4DKPS7B
1A4DSPM7B
1A4DKPSno6B7B
1A4DKPS4DSPMno6B7B
1AKPS4DSPMno6B7B
1ASPM4DKPSno6B7B
1A4DSPMno6B7B
(E)
Comparisons of QTL effects of KPS_17CVI
30.0
25.0
20.0
15.0
10.0
5.0
0.0
1A4DKPS6B
1AKPS6B
1ASPM4DKPS6B
1AKPS4DSPM6B
1A4DSPM6B
1A4DKPS6B7B
1AKPS6B7B
1ASPM4DKPS6B7B
1ASPM4DKPSSPM6B7B
1A4DSPM6B7B
1A4DKPS7B
1AKPS7B
1AKPS4DKPSSPM7B
1AKPS4DSPM7B
1ASPM4DKPS7B
1A4DSPM7B
1A4DKPSno6B7B
1A4DKPS4DSPMno6B7B
1AKPS4DSPMno6B7B
1ASPM4DKPSno6B7B
1A4DSPMno6B7B
(F)
Comparisons of QTL effects of KPS_17DMS
36.0
35.0
34.0
33.0
32.0
31.0
30.0
29.0
28.0
27.0
1A4DKPS6B
1AKPS6B
1ASPM4DKPS6B
1AKPS4DSPM6B
1A4DSPM6B
1A4DKPS6B7B
1AKPS6B7B
1ASPM4DKPS6B7B
1ASPM4DKPSSPM6B7B
1A4DSPM6B7B
1A4DKPS7B
1AKPS7B
1AKPS4DKPSSPM7B
1AKPS4DSPM7B
1ASPM4DKPS7B
1A4DSPM7B
1A4DKPSno6B7B
1A4DKPS4DSPMno6B7B
1AKPS4DSPMno6B7B
1ASPM4DKPSno6B7B
1A4DSPMno6B7B

(G)

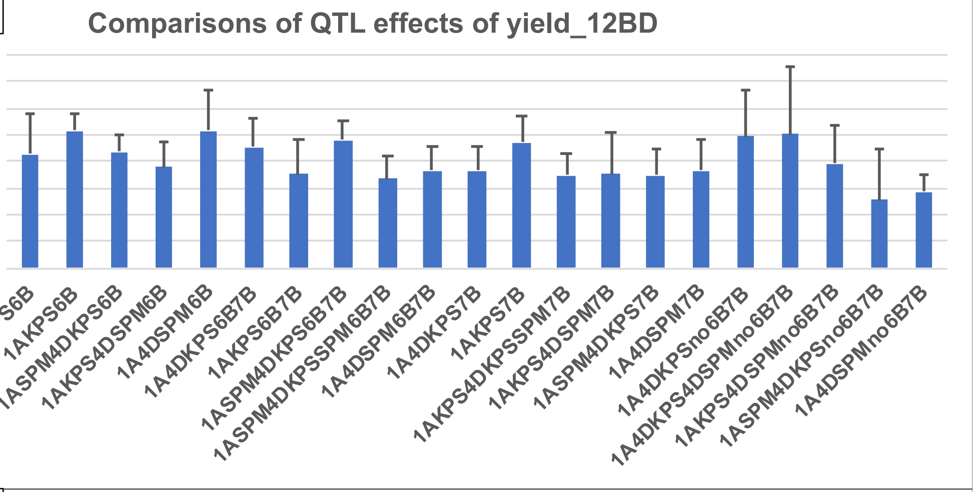
Comparisons of QTL effects of yield_12BD
175.0
170.0
165.0
160.0
155.0
150.0
145.0
140.0
135.0
1A4DKPS6B
1AKPS6B
1ASPM4DKPS6B
1AKPS4DSPM6B
1A4DSPM6B
1A4DKPS6B7B
1AKPS6B7B
1ASPM4DKPS6B7B
1ASPM4DKPSSPM6B7B
1A4DSPM6B7B
1A4DKPS7B
1AKPS7B
1AKPS4DKPSSPM7B
1AKPS4DSPM7B
1ASPM4DKPS7B
1A4DSPM7B
1A4DKPSno6B7B
1A4DKPS4DSPMno6B7B
1AKPS4DSPMno6B7B
1ASPM4DKPSno6B7B
1A4DSPMno6B7B

(H)

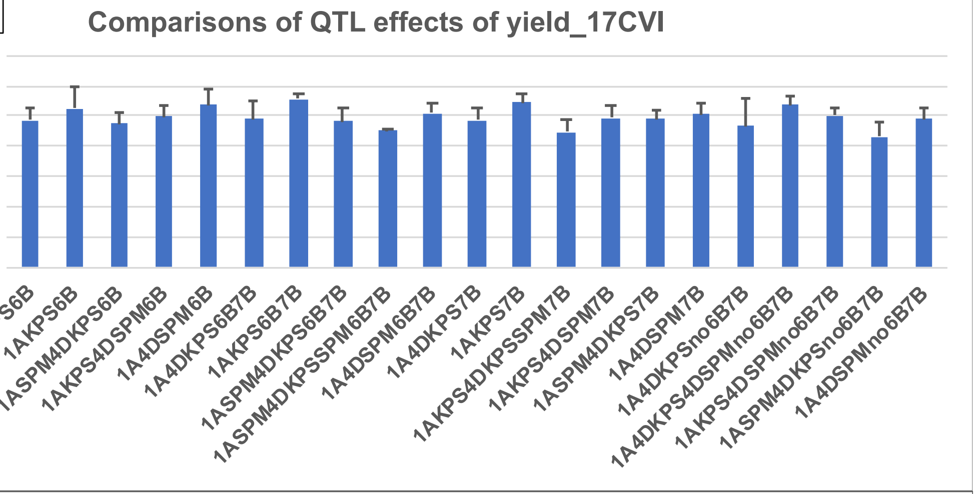
Comparisons of QTL effects of yield_17CVI
700.0
600.0
500.0
400.0
300.0
200.0
100.0
0.0
1A4DKPS6B
1AKPS6B
1ASPM4DKPS6B
1AKPS4DSPM6B
1A4DSPM6B
1A4DKPS6B7B
1AKPS6B7B
1ASPM4DKPS6B7B
1ASPM4DKPSSPM6B7B
1A4DSPM6B7B
1A4DKPS7B
1AKPS7B
1AKPS4DKPSSPM7B
1AKPS4DSPM7B
1ASPM4DKPS7B
1A4DSPM7B
1A4DKPSno6B7B
1A4DKPS4DSPMno6B7B
1AKPS4DSPMno6B7B
1ASPM4DKPSno6B7B
1A4DSPMno6B7B

(I)

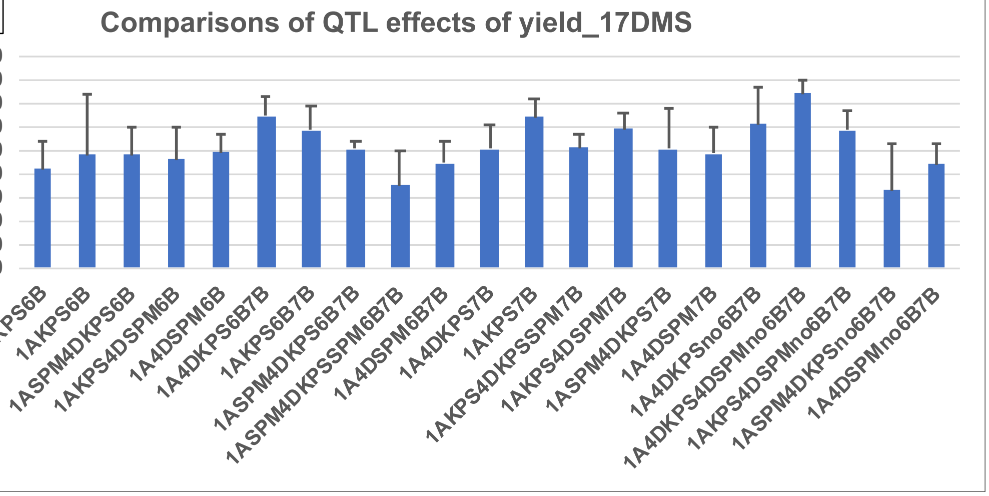
Comparisons of QTL effects of yield_17DMS
660.0
640.0
620.0
600.0
580.0
560.0
540.0
520.0
500.0
480.0
1A4DKPS6B
1AKPS6B
1ASPM4DKPS6B
1AKPS4DSPM6B
1A4DSPM6B
1A4DKPS6B7B
1AKPS6B7B
1ASPM4DKPS6B7B
1ASPM4DKPSSPM6B7B
1A4DSPM6B7B
1A4DKPS7B
1AKPS7B
1AKPS4DKPSSPM7B
1AKPS4DSPM7B
1ASPM4DKPS7B
1A4DSPM7B
1A4DKPSno6B7B
1A4DKPS4DSPMno6B7B
1AKPS4DSPMno6B7B
1ASPM4DKPSno6B7B
1A4DSPMno6B7B

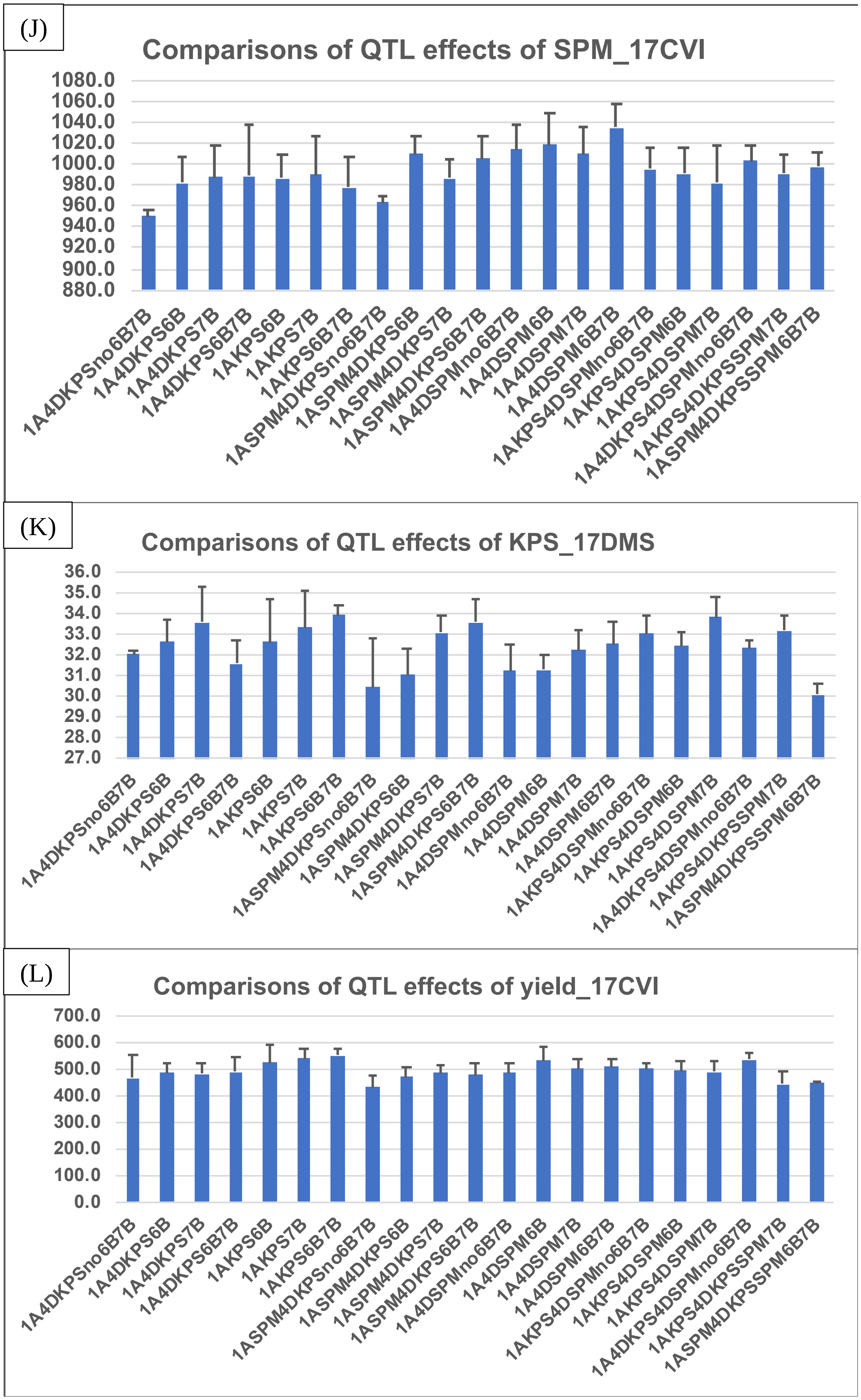
(J)
Comparisons of QTL effects of SPM_17CVI
1080.0
1060.0
1040.0
1020.0
1000.0
980.0
960.0
940.0
920.0
900.0
880.0
1A4DKPSno6B7B
1A4DKPS6B
1A4DKPS7B
1A4DKPS6B7B
1AKPS6B
1AKPS7B
1AKPS6B7B
1ASPM4DKPSno6B7B
1ASPM4DKPS6B
1ASPM4DKPS7B
1ASPM4DKPS6B7B
1A4DSPMno6B7B
1A4DSPM6B
1A4DSPM7B
1A4DSPM6B7B
1AKPS4DSPMno6B7B
1AKPS4DSPM6B
1AKPS4DSPM7B
1A4DKPS4DSPMno6B7B
1AKPS4DKPSSPM7B
1ASPM4DKPSSPM6B7B
(K)
Comparisons of QTL effects of KPS_17DMS
36.0
35.0
34.0
33.0
32.0
31.0
30.0
29.0
28.0
27.0
1A4DKPSno6B7B
1A4DKPS6B
1A4DKPS7B
1A4DKPS6B7B
1AKPS6B
1AKPS7B
1AKPS6B7B
1ASPM4DKPSno6B7B
1ASPM4DKPS6B
1ASPM4DKPS7B
1ASPM4DKPS6B7B
1A4DSPMno6B7B
1A4DSPM6B
1A4DSPM7B
1A4DSPM6B7B
1AKPS4DSPMno6B7B
1AKPS4DSPM6B
1AKPS4DSPM7B
1A4DKPS4DSPMno6B7B
1AKPS4DKPSSPM7B
1ASPM4DKPSSPM6B7B
(L)
Comparisons of QTL effects of yield_17CVI
700.0
600.0
500.0
400.0
300.0
200.0
100.0
0.0
1A4DKPSno6B7B
1A4DKPS6B
1A4DKPS7B
1A4DKPS6B7B
1AKPS6B
1AKPS7B
1AKPS6B7B
1ASPM4DKPSno6B7B
1ASPM4DKPS6B
1ASPM4DKPS7B
1ASPM4DKPS6B7B
1A4DSPMno6B7B
1A4DSPM6B
1A4DSPM7B
1A4DSPM6B7B
1AKPS4DSPMno6B7B
1AKPS4DSPM6B
1AKPS4DSPM7B
1A4DKPS4DSPMno6B7B
1AKPS4DKPSSPM7B
1ASPM4DKPSSPM6B7B
