# Supplementary material for: Genome wide identification of QTL associated with yield and yield components in two popular wheat cultivars TAM 111 and TAM 112
Source: PLoS One. 2020 Dec 2;15(12):e0237293. doi: 10.1371/journal.pone.0237293 (PMC7710072; doi:10.1371/journal.pone.0237293)

S5 Fig. Whole genome significance profiles of epistasis at LOD > 10 for yield and its components. The numbers on the rings showing the peak cM position of the target SNPs on that chromosome. The numbers on each line showing the total LOD score of that epistasis. Detailed information of SNP and its corresponding chromosome and cM locations, LOD scores and percentage of variations explained for total, additive and additive by environments, additive effects for 1st and 2nd QTL linked SNPs, their corresponding additive by environment, epistasis by environment were list in Table S5.

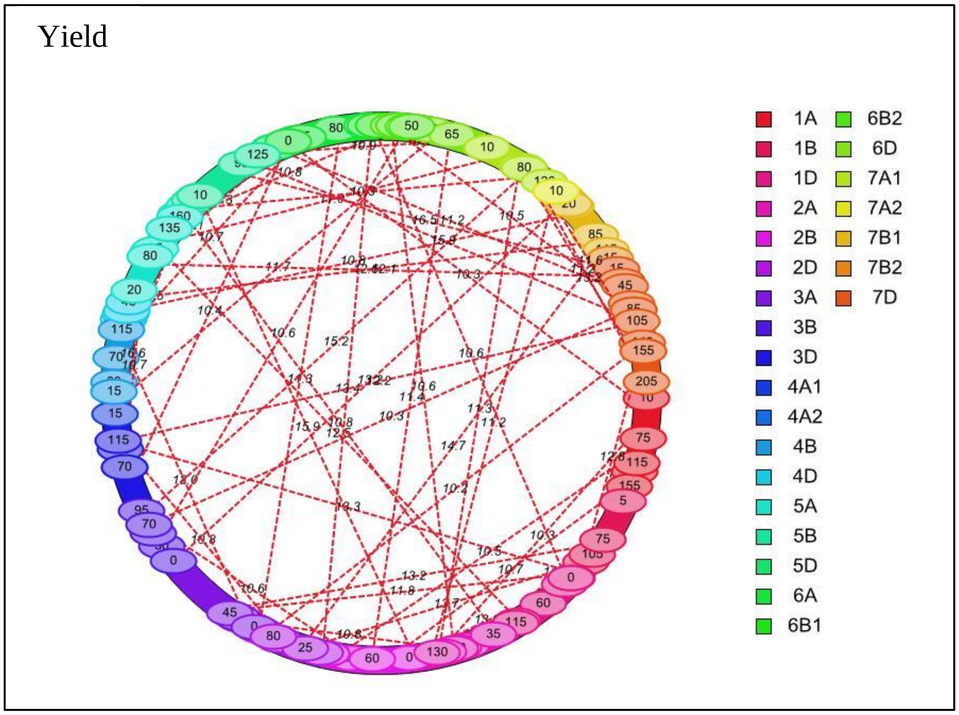

Harvest Index

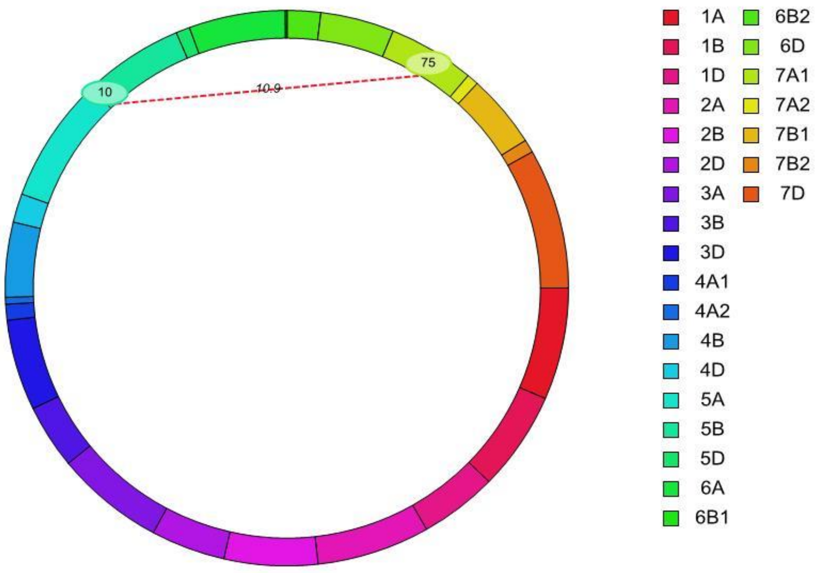

Kernels spike^{-1}

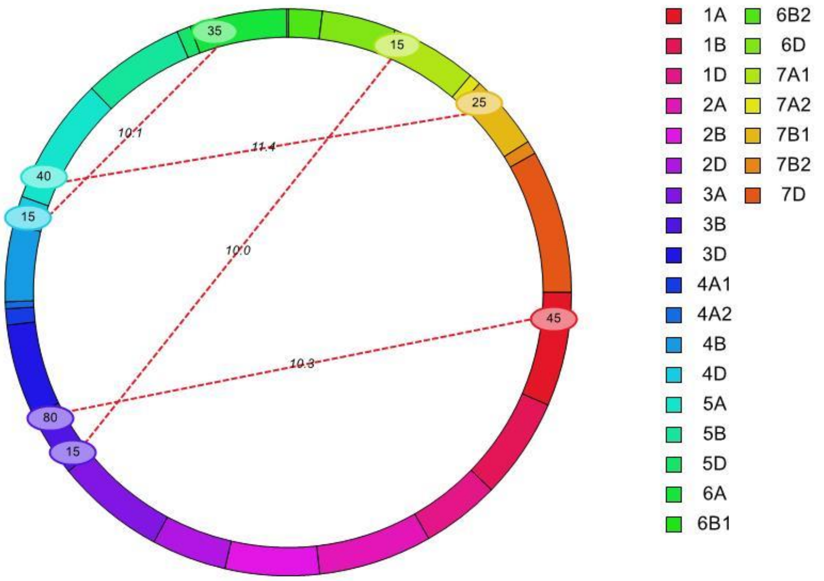

Spikes m^{-2}

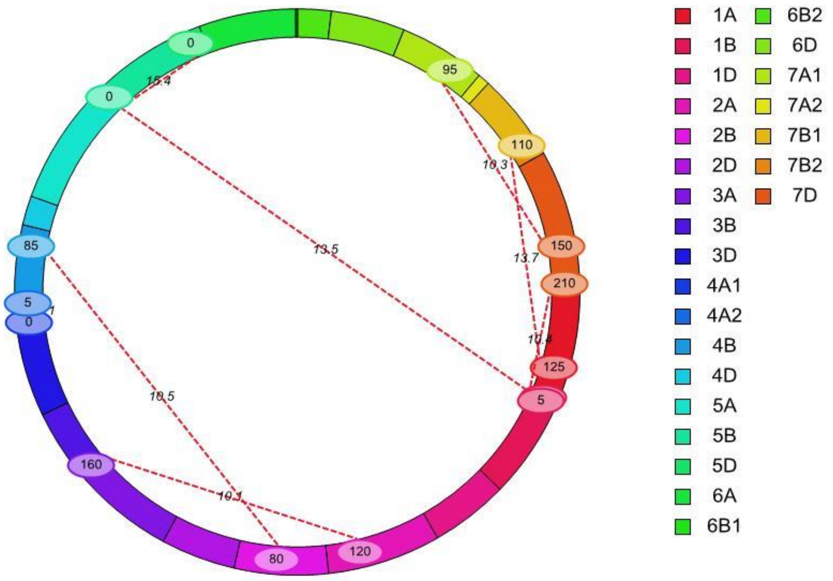

Thousand kernel weight

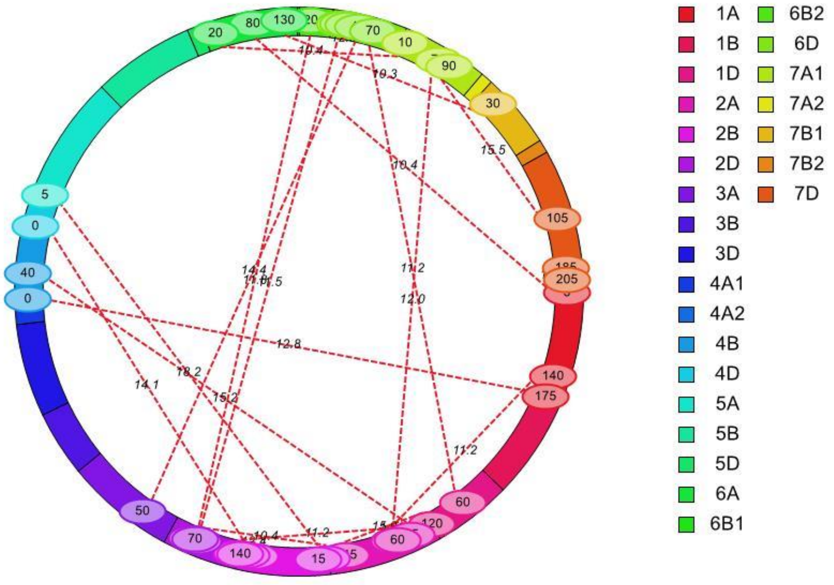

Supplement: S5 Fig — (PDF) [file pone.0237293.s005.pdf]
